# Overexpression of ameloblastin in secretory ameloblasts results in demarcated, hypomineralized opacities in enamel

**DOI:** 10.3389/fphys.2023.1233391

**Published:** 2024-01-11

**Authors:** Yong-Hee Patricia Chun, Chunyan Tan, Omar Villanueva, Madeline E. Colley, Travis J. Quintanilla, Mohamed S. Basiouny, Caldonia A. Hartel, Cameron S. Critchfield, Stephan B. H. Bach, Roberto J. Fajardo, Cong-Dat Pham

**Affiliations:** ^1^ Department of Periodontics, School of Dentistry, University of Texas Health Science Center at San Antonio, San Antonio, TX, United States; ^2^ Department of Cell Systems and Anatomy, School of Medicine, University of Texas Health Science Center at San Antonio, San Antonio, TX, United States; ^3^ Department of Molecular Medicine, School of Medicine, University of Texas Health Science Center at San Antonio, San Antonio, TX, United States; ^4^ Department of Chemistry, University of Texas San Antonio, San Antonio, TX, United States; ^5^ Department of Biochemistry, Vanderbilt University, Nashville, TN, United States; ^6^ Mass Spectrometry Research Center, Vanderbilt University, Nashville, TN, United States; ^7^ Department of Clinical and Applied Science Education, School of Osteopathic Medicine, University of the Incarnate Word, San Antonio, TX, United States; ^8^ Department of Cancer Systems Imaging, University of Texas MD Anderson Cancer Center, Houston, TX, United States

**Keywords:** molar–incisor hypomineralization, enamel hypomineralization, enamel demarcation, enamel formation, mineralization, ameloblastin (Ambn), secreted calcium-binding phosphoproteins (SCPPs)

## Abstract

**Introduction:** Developmental defects of the enamel manifest before tooth eruption and include amelogenesis imperfecta, a rare disease of underlying gene mutations, and molar–incisor hypomineralization (MIH), a prevalent disease in children originating from environmental and epigenetic factors. MIH enamel presents as the abnormal enamel marked by loss of translucency, demarcation between the healthy and affected enamel, and reduced mineral content. The pathophysiology of opaque, demarcated enamel lesions is not understood; however, the retention of enamel proteins in the matrix has been suggested. Ameloblastin (Ambn) is an enamel protein of the secreted calcium-binding phosphoproteins (SCPPs) critical for enamel formation. When the *Ambn* gene is mutated or deleted, teeth are affected by hypoplastic amelogenesis imperfecta.

**Methods:** In this study, enamel formation in mice was analyzed when transgenic *Ambn* was overexpressed from the amelogenin promoter encoding full-length Ambn. *Ambn* was under- and overexpressed at six increasing concentrations in separate mouse lines.

**Results:** Mice overexpressing *Ambn* displayed opaque enamel at low concentrations and demarcated lesions at high concentrations. The severity of enamel lesions increased starting from the inner enamel close to the dentino-enamel junction (DEJ) to span the entire width of the enamel layer in demarcated areas. Associated with the opaque enamel were 17-kDa Ambn cleavage products, a prolonged secretory stage, and a thin basement membrane in the maturation stage. Ambn accumulations found in the innermost enamel close to the DEJ and the mineralization front correlated with reduced mineral content. Demarcated enamel lesions were associated with Ambn species of 17 kDa and higher, prolonged secretory and transition stages, a thin basement membrane, and shortened maturation stages. Hypomineralized opacities were delineated against the surrounding mineralized enamel and adjacent to ameloblasts detached from the enamel surface. Inefficient Ambn cleavage, loss of contact between ameloblasts, and the altered basement membrane curtailed the endocytic activity; thus, enamel proteins remained unresorbed in the matrix. Ameloblasts have the ability to distinguish between Ambn concentration and Ambn cleavage products through finely tuned feedback mechanisms. The under- or overexpression of Ambn in murine secretory ameloblasts results in either hypoplastic amelogenesis imperfecta or hypomineralization with opaque or sharply demarcated boundaries of lesions, similar to MIH.

## Introduction

Hypomineralized enamel presents as a developmental defect of enamel formation (Clarkson and O’Mullane, 1989; Suga, 1989). It gained attention as molar–incisor hypomineralization (MIH) in dentistry, since the introduction of this term in 2001 at the annual conference of the European Academy of Pediatric Dentistry (Weerheijm et al., 2001). Prior to consensus on the term “MIH,” the hypomineralized enamel had been described as “idiopathic enamel hypomineralization” or “enamel hypomineralization of first permanent molars” (Koch et al., 1987; Jalevik and Noren, 2000), “nonfluoride hypomineralization” or “demarcated hypomineralization” (Holtta et al., 2001; Leppaniemi et al., 2001), and “cheese molars” (Van Amerongen and Kreulen, 1995). Most recently, “molar hypomineralization” was proposed to acknowledge that molars are most prominently affected (Hubbard et al., 2017). For this report, MIH was selected as a term for its longstanding acceptance in the field.

The clinical presentation and etiology of MIH are distinct from fluorosis, enamel hypoplasia, and amelogenesis imperfecta. The distinguishing feature of MIH lesions from fluorosis is their demarcation from sound enamel such that the affected and unaffected enamel are in juxtaposition, with abrupt changes in mineral density (Clarkson and O’Mullane, 1989; Houari et al., 2023). MIH is globally prevalent in 13.5% of children (Lopes et al., 2021). In affected children, MIH enamel fails to form correctly, debilitating children with chalky, brittle patches of enamel, sensitive teeth, and increased risk for caries (Vieira et al., 2022). MIH burdens affected individuals through inefficient mastication and dysfunctional social interactions.

The underlying etiology of MIH disrupts the ameloblast function without the possibility of regaining normal function. Multiple factors of systemic, genetic, and epigenetic origin may coincide to damage ameloblasts irreversibly (Alaluusua, 2010). Since enamel-forming ameloblasts are lost at tooth eruption, much of the current understanding of the pathophysiology of MIH was gained from the analysis of extracted teeth affected by MIH (Fagrell et al., 2010; Farah et al., 2010; Farah et al., 2010; Fagrell et al., 2013). MIH teeth are characterized by demarcated discoloration, ranging from white through yellow to brown, reduced hardness, reduced mineral density, and increased protein content, paired with rapid post-eruptive enamel breakdown. Recently, the analysis of the MIH enamel identified serum albumin and other blood proteins (Perez et al., 2020; Farah et al., 2010, Mangum et al., 2010), proposing albumin as an impediment to the growth of enamel crystals and attainment of normal mineral content (Hubbard et al., 2021). However, cellular events of the pathophysiology are still unknown, and the need for understanding the pathways leading to opaque, demarcated enamel lesions has been pointed out (Zameer and Birajdar, 2022). Animal models induced opaque hypomineralized enamel with bisphenol A and amoxicillin in rats (Jedeon et al., 2013; Duman et al., 2022); demarcated lesions were induced by infections with parasites in sheep (Suckling et al., 1983; Suckling et al., 1986).

In this study, we propose a mouse model for opaque, demarcated, hypomineralized enamel that originates from the overexpression of the enamel protein ameloblastin (Ambn) encoded as a full-length protein in the secretory stage of amelogenesis. Ambn expression was increased in four separate mouse lines, resulting in a spectrum of opaque, hypomineralized enamel. Ambn is an essential enamel protein causing amelogenesis imperfecta when the gene is deleted or mutated in mice or humans (Poulter et al., 2014; Liang et al., 2019). *Ambn* is a gene within the secreted calcium-binding phosphoprotein (SCPP) cluster on chromosome 4 in humans and chromosome 5 in mice (Kawasaki and Weiss, 2008). Genes of the SCPP duplicated from SPARCL1 and the ancestral gene SPARC (Kawasaki et al., 2004). SPARC is a component of the dermal skeleton and initiates mineralization through calcium-binding abilities. Similar to other SCPPs, the Ambn structure is intrinsically disordered, cleaved into peptides by matrix metalloproteinase (MMP) 20, and modified by post-translational phosphorylations, O-linked glycosylations, and hydroxylations (Yamakoshi et al., 2001; Kobayashi et al., 2007; Chun et al., 2010; Su et al., 2019). Ambn peptides may carry out multiple functions in enamel mineralization, cell adhesion, and removal of degraded enamel proteins (Uchida et al., 1998; Fukumoto et al., 2004).

In this first report of Ambn overexpression linked to demarcated enamel hypomineralization, the goal of this study was to characterize ameloblast and enamel organ morphology, localize Ambn protein, and highlight pathways for protein retention.

## Materials and methods

### Animals

The use and care of animals adhered to the guidelines of the National Academy of Sciences. Animal protocols were reviewed and approved by the Institutional Animal Care and Use Committee of UT Health San Antonio (UTHSA). Mice expressing full-length *Ambn* transgenically (Tg) from the promoter of the *AmelX* gene were mated with wild-type mice to generate mice overexpressing *Ambn* at four increasing concentrations, from low to very high (Tg^+^, ^++^, ^+++^, and ^+++/+++^), in the C57BL6 genetic background (Chun et al., 2010). *Ambn* ^*Δ5,6/Δ5,6*^ mice lacking exons 5 and 6 (Ambn^Δ5,6/Δ5,6^) display amelogenesis imperfecta and represent *Ambn* underexpression (Fukumoto et al., 2004). The inclusion of six *Ambn* genotypes (Ambn^Δ5,6/Δ5,6^, wild-type, and four Ambn Tg concentrations) presents a series of alleles from non-functional to multiple copies. The importance of the correct concentration of Ambn was recognized from the under- or overexpression of homologous SPARC, both resulting in a dysregulated matrix (Trombetta-Esilva and Bradshaw, 2012). Genotyping was conducted from tail snips, as reported previously (Chun et al., 2010). Mice were housed in the UTHSA husbandry with unlimited access to soft diet and water.

### Real-time quantitative PCR

For each genotype, enamel organs of the first mandibular molars were dissected on dry ice from postnatal day 5 mice and homogenized with TRIzol (Thermo Fisher Scientific, Carlsbad, CA, USA). Chloroform was added, and the phases were separated by centrifuging. Total RNA was precipitated by adding isopropanol and reverse-transcribed. qPCR reactions with primers for *Ambn* 5′UTR (forward aag​tgt​cag​cac​ttg​gtg​gt and reverse tca​cat​ttc​ctg​ggc​ata​at), *Ambn* Tg (forward act​caa​aga​acc​atc​aag​GG and reverse ccc​agg​ttg​ttg​agg​aaa​tg), and *GAPDH* (forward tga​cgt​gcc​gcc​tgg​aga​aa and reverse agt​gta​gcc​caa​gat​gcc​ctt​cag) were conducted with SYBR Green. Relative expression levels were calculated by the 2^−ΔΔCt^ method (Livak and Schmittgen, 2001).

### SDS-PAGE and Western blot

Mandibular molars were dissected at the secretory stage (postnatal day 5). Proteins were extracted with 0.5% formic acid, lyophilized, resuspended in Laemmli buffer, and loaded on 4%–12% NuPage Bis-Tris gels (Life Technologies, Carlsbad, CA, USA) for electrophoresis. One gel was stained with Coomassie brilliant blue (Life Technologies, Carlsbad, CA, USA) and washed in water. For Western blot analysis, proteins separated on a second gel by electrophoresis were transferred onto a PVDF membrane. Membranes were then incubated with the anti-Ambn antibody at a dilution of 1:4,000 (custom-made, N-terminal) (Kobayashi et al., 2007) in 5% blocking milk in TBS-Tween. A secondary antibody with conjugated HRP was added at 1:50,000 for 1 h, and signals were detected with the ECL substrate.

### Microcomputed tomography

At the age of 7 weeks, mandibles were dissected from male and female mice, fixated in 4% PFA by intracardial perfusion, and dehydrated in 70% ethanol. Hemi-mandibles were scanned using the SkyScan 1172 (Bruker, Allentown, PA) instrument with a 0.5-mm aluminum filter at 60 kV and a beam intensity of 167 µA (Schmitz et al., 2014). The rotation step was 0.35° with a 1,090-millisecond (msec) exposure time at each step. Images were reconstructed using NRecon (SkyScan, Aartselaar, Belgium) with a modified Feldkamp cone-beam algorithm and had an image pixel size of 5 μm.

### Histology

Hemi-mandibles were trimmed and paraffin-embedded for sagittal and transverse sectioning. For staining tissue sections with Masson’s trichrome stain (Polysciences, Warrington, PA, USA; Cat # 25088-1), PFA-fixed, decalcified, and paraffin-embedded sections were baked and deparaffinized. Sections were placed in Weigert’s iron hematoxylin for 10 min, washed in running tap water, and then stained with Biebrich scarlet–acid fuchsin for 4 min and 40 s. After rinsing with water, the slides were placed in phosphotungstic/phosphomolybdic acid, drained, and transferred to an aniline blue solution. The rinsed slides were then placed in 1% acetic acid for 1 min. Tissues were dehydrated in 95% and 100% ethanol, and mounted with a xylene-based mounting medium.

### Mass spectrometry imaging

Paraffin-embedded hemi-mandibles were sectioned and placed on indium-tin-oxide-coated one-surface slides (R_s_ 5- 5–10 Ω, cut edges, Delta Technologies Ltd., Loveland, CO, USA) to facilitate conduction. The slides were washed in xylene to remove paraffin and then dehydrated. Antigen retrieval was performed with 10 mM Tris Base (pH 8.0) for 1 h at 60°C. Trypsin (0.1 μg/μL) was applied using a tissue sprayer (SunChrom, Friedrichsdorf, Germany) at 15 μL/min with 30 passes. 2,5-Dihydroxybenzoic acid (Acros Organics, NJ, USA) was applied by sublimation with a device (Chemglass Life Sciences, Vineland, NJ, USA) for 4.5 min at 20 mTorr and 145 °C sand bath. The slide was rehydrated with 2.5% acetic acid by volume for 30 s until the matrix layer changed color from powdery white to slightly opaque. MALDI-TOF mass spectrometry was used with Flex Imaging to obtain mass spectra at every 25 μm pixel in a reflectron-positive mode, and the data were calibrated with peptide calibration standard II (Bruker Daltonics, Billerica, MA, USA). Identifications were performed on tissue by MALDI-TOF/TOF using the Bruker UltrafleXtreme LIFT module in the mass spectrometer (Bruker Daltonics, Billerica, MA, USA). The MS/MS spectra were assigned identification with Mascot through the NCBI database (Colley et al., 2020).

### Immunohistochemistry

Hemi-mandibles were sectioned transversely at six levels and immunostained using ImmPRESS (Vector Laboratories, Newark, CA). Antigen retrieval was performed in 10 mM sodium citrate-based buffer (pH 6.0), followed by equilibration in 10 mM sodium phosphate (pH 7.5). Blocking was performed with BLOXALL (Vector Laboratories) and normal horse serum (2.5%). Sections were then incubated with rabbit primary antibodies anti-Ambn (N-terminal, custom-made) at 1:30,000, anti-Rab5a (Proteintech, Rosemont, IL, Cat # 20228-1-AP) at 1:500, or anti-cathepsin D (Alpha Diagnostics, CTHD11-A) at 1:50 dilution in PBS-T. Rab5 localizes to early endosomes; cathepsin D localizes to lysosomes (Pham et al., 2017). Sections were incubated in ImmPRESS Polymer Reagent, washed, and incubated in ImmPACT DAB. Sections were counterstained with methyl green or hematoxylin, rinsed, and mounted, and images were then taken.

## Results

### Macroscopic phenotype of incisors and molars with overexpressed Ambn

**FIGURE 1:**
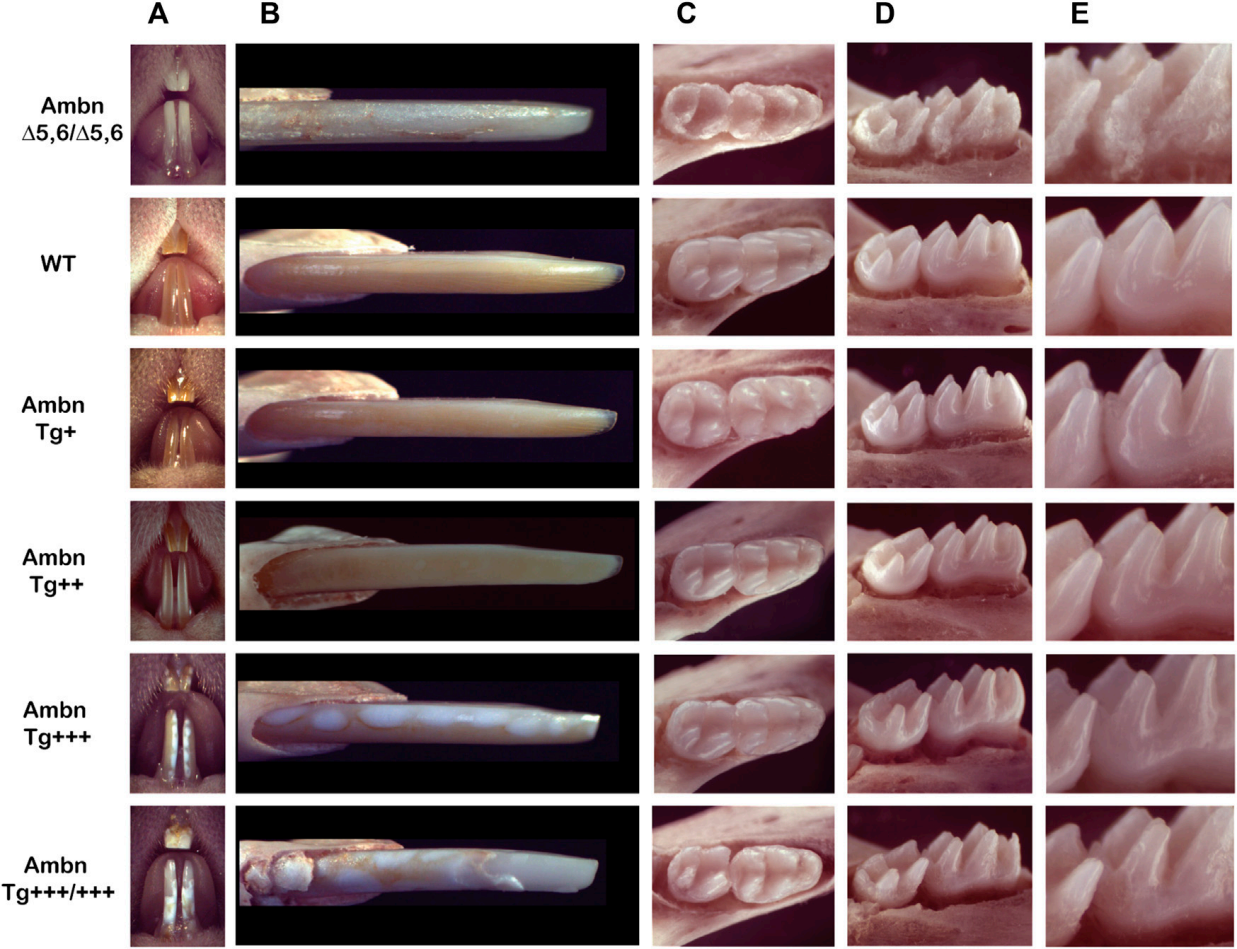
Phenotype of Ambn under- and overexpression in mouse mandibular incisors and molars. **(A)** The frontal view of maxillary and mandibular incisors shows the enamel color, contour, and surface textures in the absence of functional Ambn (*Ambn* ^*Δ5,6/Δ5,6*^), or overexpression of Ambn (Tg) at four concentrations. **(B)** The close view of the erupted incisor enamel demonstrates a correlation of phenotype severity with the *Ambn Tg* genotype. In *Ambn Tg* ^*+*^ incisors, no changes in the enamel color or texture were noted compared to the wild-type (WT). In *Ambn Tg* ^*++*^ incisors, the enamel color was less orange, opaque, and the surface texture was matte instead of glossy. White, demarcated patches of regular sizes in *Ambn Tg* ^*+++*^ and irregular sizes with delamination of the enamel from the incisal edge in *Ambn Tg* ^*+++/+++*^ genotypes were noticed. Ectopic mineralization was found close to the alveolar bone at the eruption site of *Ambn Tg* ^*+++/+++*^. *Ambn* ^*Δ5,6/Δ5,6*^ enamel demonstrated hypoplastic amelogenesis imperfecta. **(C)** At postnatal day 18, the first and second molars erupted into the oral cavity. Occlusal views of mandibular molars show a reduced enamel volume in *Ambn Tg* ^*+++*^ and *Ambn Tg* ^*+++/+++*^ animals, noted in the lack of an emergence profile and thin slopes of cusps. *Ambn Tg* ^*+++/+++*^ animals had extensive surface roughness and irregularities on the slopes of cusps. The periodontal ligament was widened adjacent to the thin alveolar bone. **(D)** The enamel of molars of *Ambn Tg* ^*++*^ animals was comparable to wild-type in volume and surface texture. *Ambn Tg* ^*+++*^ and *Ambn Tg* ^*+++/+++*^ animals had a rough enamel and appeared less voluminous on the cusp slopes. The alveolar bone surrounding the mandibular molars of *Ambn Tg* ^*+++*^ animals was more brittle compared to WT. In *Ambn Tg* ^*+++*^ and *Ambn Tg* ^*+++/+++*^ animals, the crest of the alveolar bone was more apically positioned on the first and second molars. **(E)** The increase in *Ambn Tg* resulted in the loss of cusp volume and slender appearance of cusps. In the first molar of *Ambn Tg* ^*+++/+++*^, dentin was exposed on the distobuccal cusp.

Incisors under- or overexpressing *Ambn* exhibited chalky enamel with gradual development of distinct manifestations (Figure 1). Enamel is absent in *Ambn* ^*Δ5,6/Δ5,6*^ mice, except for residual deposits, resulting in a rough surface, the most severe form of hypoplastic amelogenesis imperfecta (Fukumoto et al., 2004; Chun et al., 2010). Mice overexpressing *Ambn* had chalky patches of enamel, which increased in size when the Ambn concentration increased. The two lower concentrations of *Ambn* (*Tg* ^*+*^ and *Tg* ^*++*^) had similarly colored enamel to wild-type enamel. *Ambn Tg* ^*++*^ appeared slightly less orange with a greyish hue and opaque appearance (Figure 1A). Small round, white spots were visible at a closer view (Figure 1B). Mice expressing *Ambn Tg* ^*+++*^ or *Tg* ^*+++/+++*^ had delineated white opacities. In *Ambn Tg* ^*+++*^ animals, lesions were round, similar in size, and surrounded by pigmented enamel. The lesions followed the length of the incisor. The enamel with *Ambn Tg* ^*+++/+++*^ expression had irregularly sized, coalescing white lesions and delaminated at the incisal tip.

Mouse molars lacked the orange iron pigment which is only present in incisors in outer enamel and is more acid-resistant than the inner enamel (Heap et al., 1983; Møinichen et al., 1996). Mandibular molars of *Ambn Tg* ^*+++*^ animals were flatter in their overall dimensions and had a reduced emergence profile due to volumetric deficiencies (Figures 1C–E). Enamel at the slopes of the cusps, lingual, buccal, and interproximal surfaces was uneven and lacked smoothness, a hallmark of wild-type enamel.

### Ameloblastin expression in the enamel organ epithelium and enamel matrix

**FIGURE 2:**
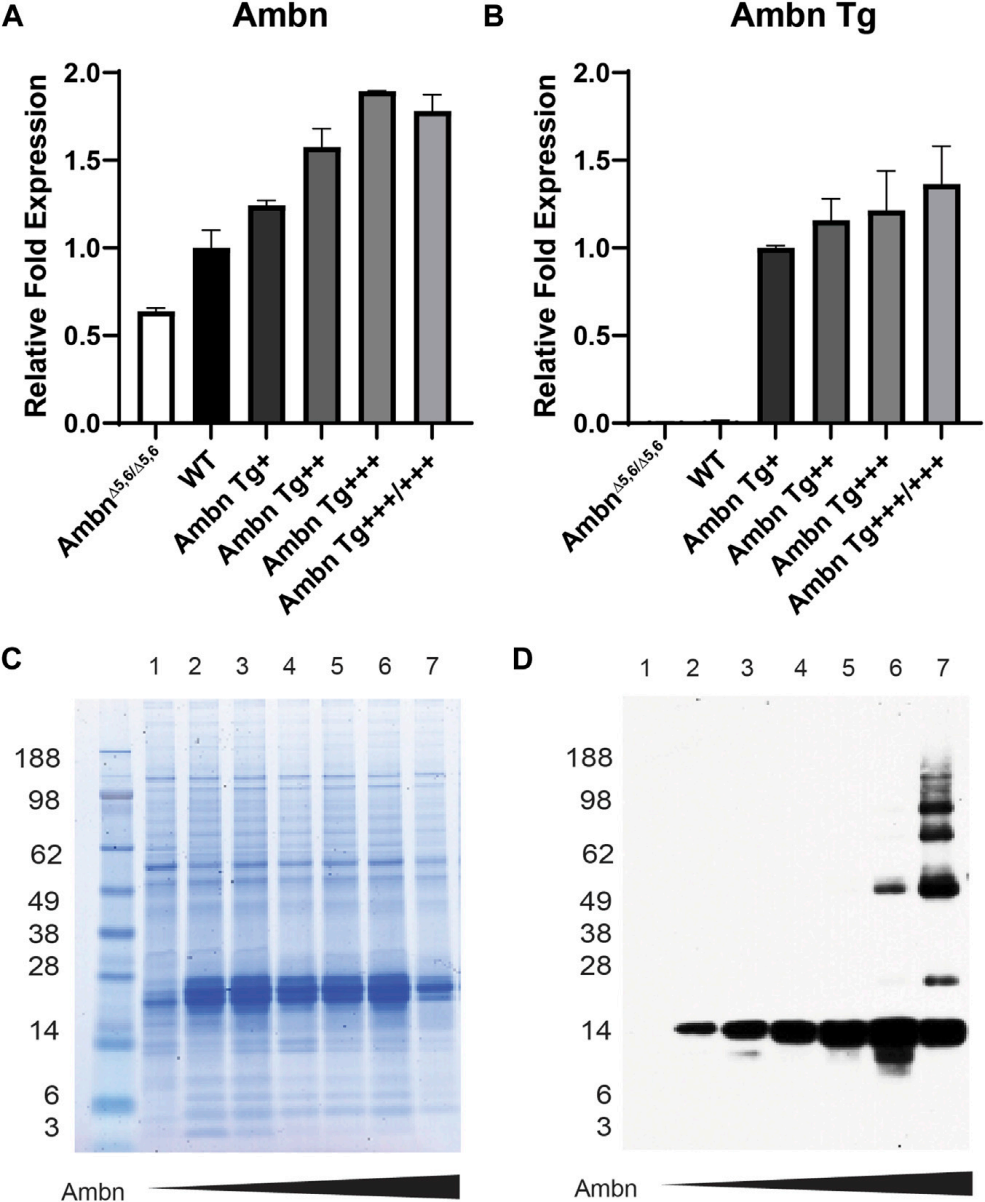
Ambn expression in the enamel organ epithelium and molars. Enamel organ epithelium and first mandibular molars of 5-day-old mice were used. **(A)** qPCR demonstrated the proportional increase in *Ambn* 5′ UTR expression in the enamel organ epithelium at the secretory stage in *Ambn Tg* animals compared to wild-type and *Ambn* ^*Δ5,6/Δ5,6*^. The enamel organ epithelium of three animals was analyzed. Statistical analysis with ANOVA revealed differences between wild-type and each genotype. **(B)** qPCR was conducted with primers specifically amplifying the transgene of Ambn. The relative increase in Ambn expression from *Ambn Tg* ^*+*^, ^*++*^ *,* ^*+++*^ *,* and ^*+++/+++*^ was demonstrated. Statistical analysis with ANOVA revealed differences between *Ambn Tg* ^*+*^ and each genotype. **(C)** The CBB-stained gel showed even loading of extracted enamel proteins. In *Ambn* ^*Δ5,6/Δ5,6*^ and the highest *Ambn Tg* expresser, enamel proteins were reduced, suggesting that Ambn expression downregulated other enamel proteins. **(D)** The Western blot analyzed Ambn expression as an increase in concentration in the 17-kDa Ambn cleavage product. In *Ambn Tg* ^*+++*^ and *Ambn Tg* ^*+++/+++*^, molecular weight species of 50 kDa and higher appeared, suggesting inefficient enzymatic activity of enamel proteinases. Lanes: 1, Ambn^Δ5,6/Δ5,6^; 2, Ambn^5,6+/Δ5,6^; 3, WT; 4, Ambn Tg^+^; 5, Ambn Tg^++^; 6, Ambn Tg^+++^; 7, Ambn Tg^+++/+++^.

The enamel organ epithelium expressed *Ambn* mRNA in increasing concentrations from *Ambn* 5′UTR (Figure 2A). The expression of Ambn was slightly reduced in *Ambn Tg* ^*+++/+++*^ compared to *Ambn Tg* ^*+++*^ animals. *Ambn Tg*-specific expression was incrementally increased in four separate *Ambn Tg* mouse lines (Figure 2B). *Ambn Tg* was undetectable in *Ambn* ^*Δ5,6/Δ5,6*^ and the wild-type enamel organ epithelium (Figure 2B).

Full-length Ambn is known to be cleaved by Mmp20 into discrete cleavage products (Chun et al., 2010). Enamel proteins were robustly expressed in *Ambn Tg* animals (Figures 2C, D) but less abundant in the highest *Ambn Tg* line (Tg^+++/+++^) and *Ambn* ^*Δ5,6/Δ5,6*^ mice. Ambn was detected at 17 kDa in the wild-type line and all *Ambn Tg* lines. In *Ambn Tg* ^*+++*^ and *Ambn Tg* ^*+++/+++*^ teeth, an additional Ambn species was noted at 50 kDa. Furthermore, in *Ambn Tg* ^*+++/+++*^ teeth, Ambn species were 66 and 85 kDa and two species above 98 kDa (Figure 2D). Inefficient cleavage of Ambn quantity during the secretory stage could result from substrate quantity exceeding the enzymatic capacity of Mmp20.

### Hypomineralized enamel adjacent to the dentino-enamel junction and in demarcated lesions

**FIGURE 3:**
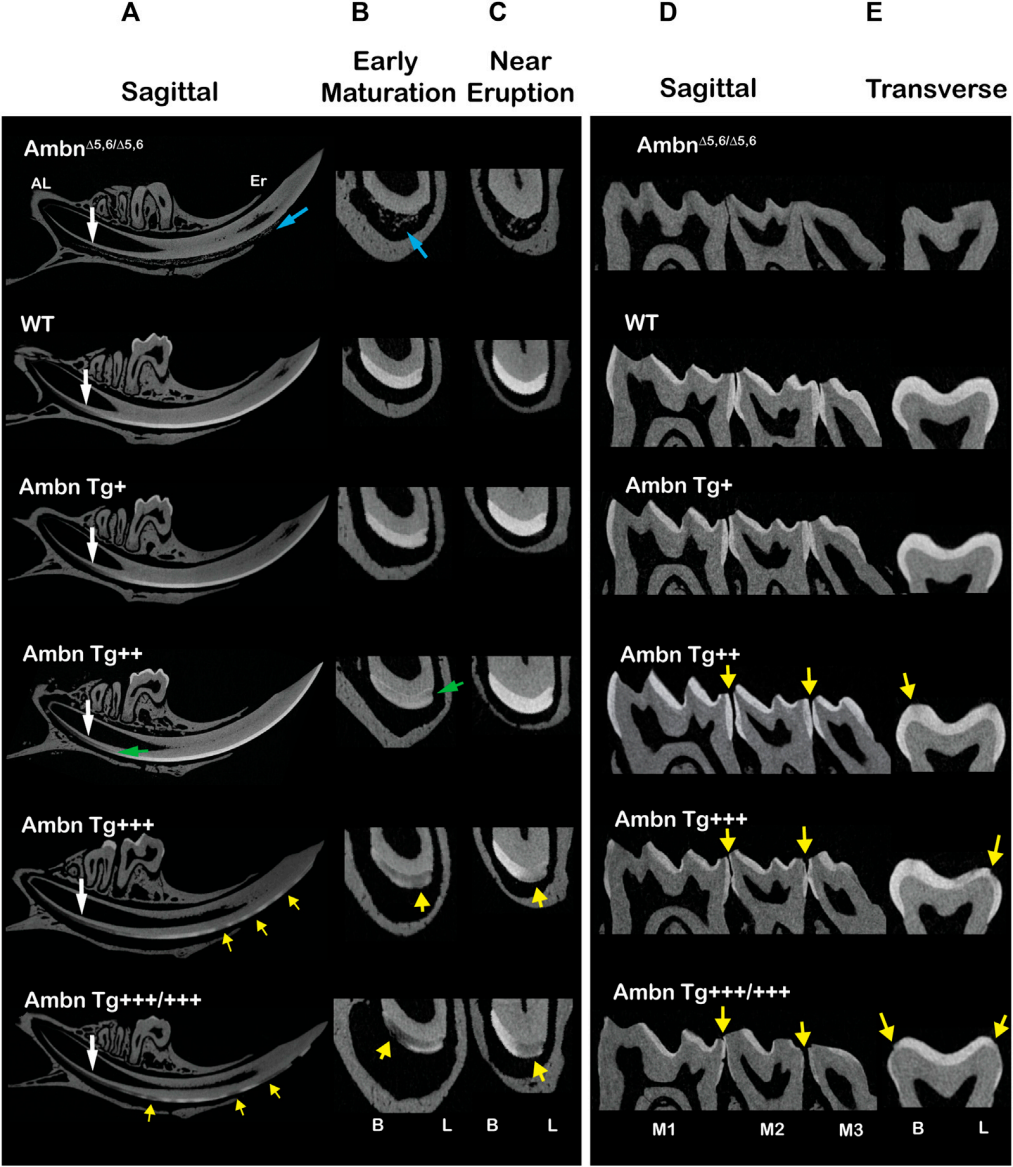
Microcomputed tomography of incisors and molars with under- or overexpressed Ambn. Incisors of 7-week-old animals were analyzed in sagittal and transverse planes. **(A)** The sagittally sliced incisor displayed enamel formation from the beginning in the apical loop (AL) to the tooth eruption (Er). *Ambn* ^*Δ5,6/Δ5,6*^ animals had ectopic minerals in the enamel organ (blue arrow) but no enamel. The timing of enamel formation was delayed in Ambn Tg^+++^ and *Ambn Tg* ^*+++/+++*^ relative to the transition from secretory to maturation stages (white arrow). *Ambn Tg* ^*++*^ incisors harbored reduced mineral density at the dentino-enamel junction (DEJ) (green arrow). *Ambn Tg* ^*+++*^ and *Ambn Tg* ^*+++/+++*^ incisors displayed hypomineralized enamel lesions alternating with mineralized enamel (yellow arrows). **(B)** Transversely sliced incisors between the second molars marked the early maturation stage of enamel formation. In *Ambn Tg* ^*++*^ incisors, the enamel was less mineralized, particularly along the DEJ (green arrow). In *Ambn Tg* ^*+++*^ and *Ambn Tg* ^*+++/+++*^, the enamel was unevenly mineralized in buccal–lingual orientation and within the height of the enamel layer (yellow arrows). **(C)** Transversely sliced incisors close to the eruption site marked reduced enamel mineralization in *Ambn Tg* ^*+++*^ and *Ambn Tg* ^*+++/+++*^, pronounced in the lingual half of the incisor (yellow arrow). In contrast, the *Ambn Tg* ^*++*^ enamel appeared comparable to wild-type (WT) in the mineral content. **(D, E)** Molars of 7-week-old animals were analyzed in sagittal and transverse planes. **(D)** All three mandibular molars (M1, M2, and M3) were captured in the sagittal image. The severity of enamel hypomineralization on the outer surfaces increased, demonstrating larger lesion sizes and decreased enamel volumes, as *Ambn Tg* concentration increased to +++ and +++/+++ (yellow arrows). **(E)** Transversely sectioned second molars displayed hypomineralization on the slopes of buccal and lingual cusps (yellow arrow). Lesions were surrounded by the mineralized enamel. B, buccal; L, lingual.

Hemi-mandibles scanned with microCT displayed incisors overexpressing *Ambn Tg* with lesions demarcated by adjacent sound enamel and dentin (Figure 3). In continuously erupting incisors, the onset of the maturation stage was progressively delayed in correlation with the incremental increase of the Ambn Tg concentration (Figures 3A–C). *Ambn* ^*Δ5,6/Δ5,6*^ animals had ectopic mineral nodules that were detached from dentin and resided within the enamel organ epithelium. In wild-type mandibular incisors, the secretory stage changes to maturation in relationship to the contact point and root apices of the second and third molars (Smith et al., 2009). In animals expressing transgenic *Ambn*, the onset of maturation was delayed (Figures 3A, B). In transverse slices of early-maturation stage *Ambn Tg* ^*++*^ incisors, under-mineralized areas close to the dentino-enamel junction (DEJ) were noticed (Figure 3B). In *Ambn Tg* ^*+++*^ and *Tg* ^*+++/+++*^ animals, these areas were more expansive and involved the entire width of the enamel layer (Figures 3A–C). Although in *Ambn Tg* ^*++*^ the mineralization in the close-to-eruption aspect of the incisor appeared similar to the wild-type enamel, in the two highest Tg concentrations, the lesions persisted in the close-to-eruption enamel (Figures 3B, C) and were confined within the mineralized enamel.

In molars of the two highest *Ambn Tg* expressing animals, the enamel was thinner, uneven, and had patches of the hypomineralized enamel at the slopes of cusps on the occlusal and buccal/lingual surfaces (Figures 3D, E). Similar to incisors, the severity of the lesions correlated with the Ambn Tg concentration. In contrast, *Ambn* ^*Δ5,6/Δ5,6*^ molars were devoid of the enamel. The underexpression of Ambn caused severe hypoplastic enamel. The overexpression of Ambn caused hypomineralization of the innermost enamel and demarcated lesions, and partially hypoplastic enamel.

### Matrix retention deregulates enamel organ morphology and timing

**FIGURE 4:**
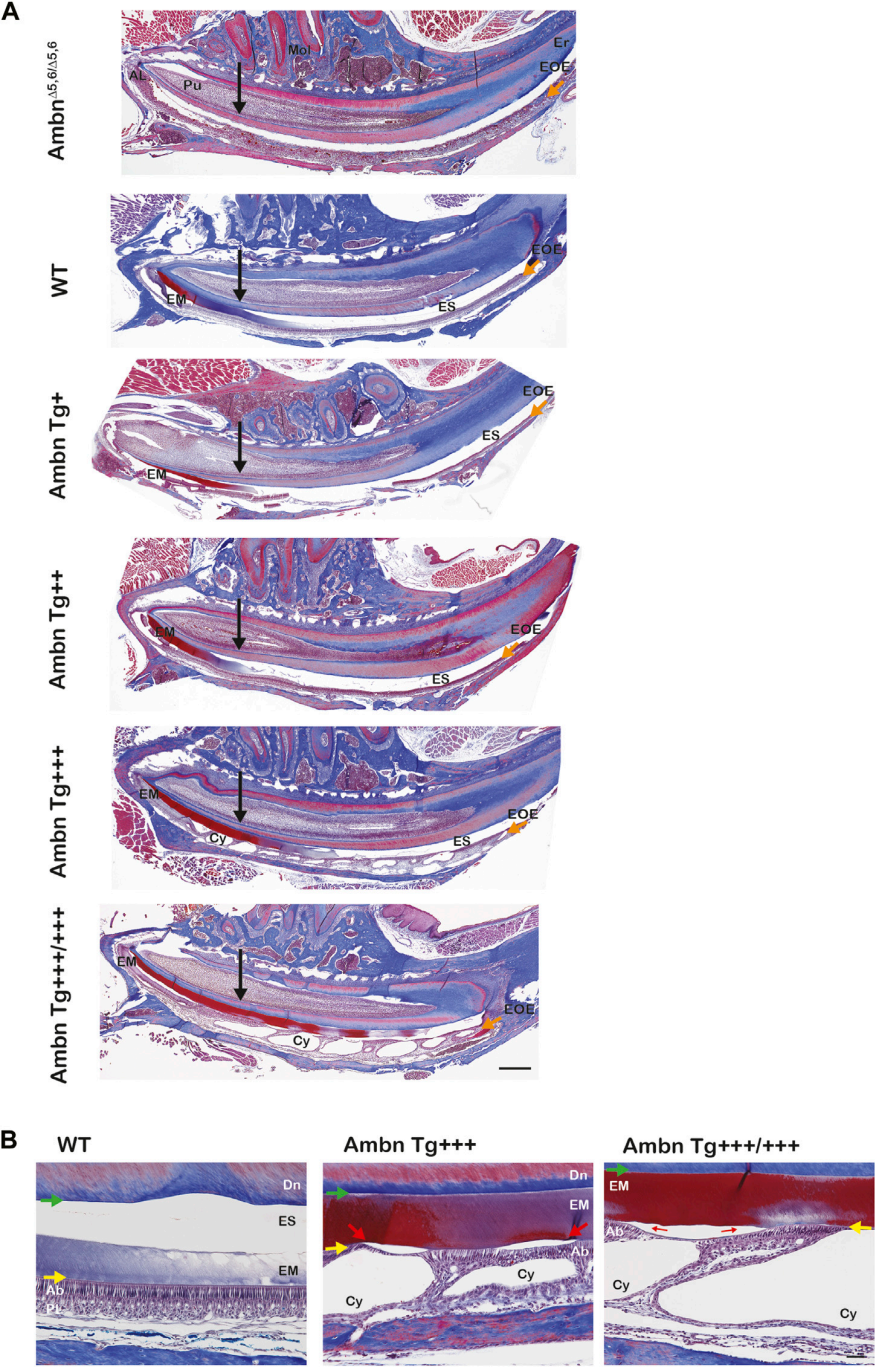
Sagittally sectioned mandibular incisors stained with Masson’s trichrome. **(A)** Hemi-mandibles were oriented with the apical loop (AL) on the left side and eruption (Er) on the right side. The switch between the secretory and maturation stages in incisors coincides with an extension of a line between the second and third mandibular molars (black arrow). The initial enamel matrix (EM) was stained red and blue before becoming degraded and resorbed in the wild-type to allow space for the enamel (ES). In animals overexpressing Ambn, the matrix was retained longer into mid-maturation stages compared to wild-type. In *Ambn Tg* ^*+++*^ and *Tg* ^*+++/+++*^ hemi-mandibles, the enamel organ exhibited cysts (Cy) with the onset of the transition stage that are present up to tooth eruption. *Ambn* ^*Δ5,6/Δ5,6*^ animals failed to deposit an enamel matrix. Scale bar, 500 μm. **(B)** Magnifications of early maturation stage ameloblasts. In wild-type (WT) animals, columnar ameloblasts are attached to the enamel surface (yellow arrow). In *Ambn Tg* ^*+++*^ incisors, cysts (Cy) were found within the enamel organ. Relative to cysts, superiorly positioned ameloblasts were flattened and detached, thereby separating ameloblasts from the enamel surface (red arrow). The enamel matrix adjacent to detached ameloblasts was more proteinaceous (red stain) than the matrix adjacent to attached ameloblasts (purple stain). The retained matrix was continuous from the dentino-enamel junction (DEJ) to the enamel surface (green arrow). The interface between the enamel surface and ameloblasts marked the basement membrane location (yellow arrow). When *Ambn Tg* ^*+++/+++*^ was expressed, the enamel matrix was retained through the thickness of the enamel layer (red stain) adjacent to detached ameloblasts. In areas with attached ameloblasts, the bulk of the enamel matrix was reduced (purple stain). AL, apical loop; Pu, pulp; Mol, molars; Er, eruption point, EOE, enamel organ epithelium; EM, enamel matrix; ES, enamel space; Er, eruption point; Ab, ameloblast; PL, papillary layer; Cy, cyst; Dn, dentin. Scale bar: 50 μm.

**FIGURE 5:**
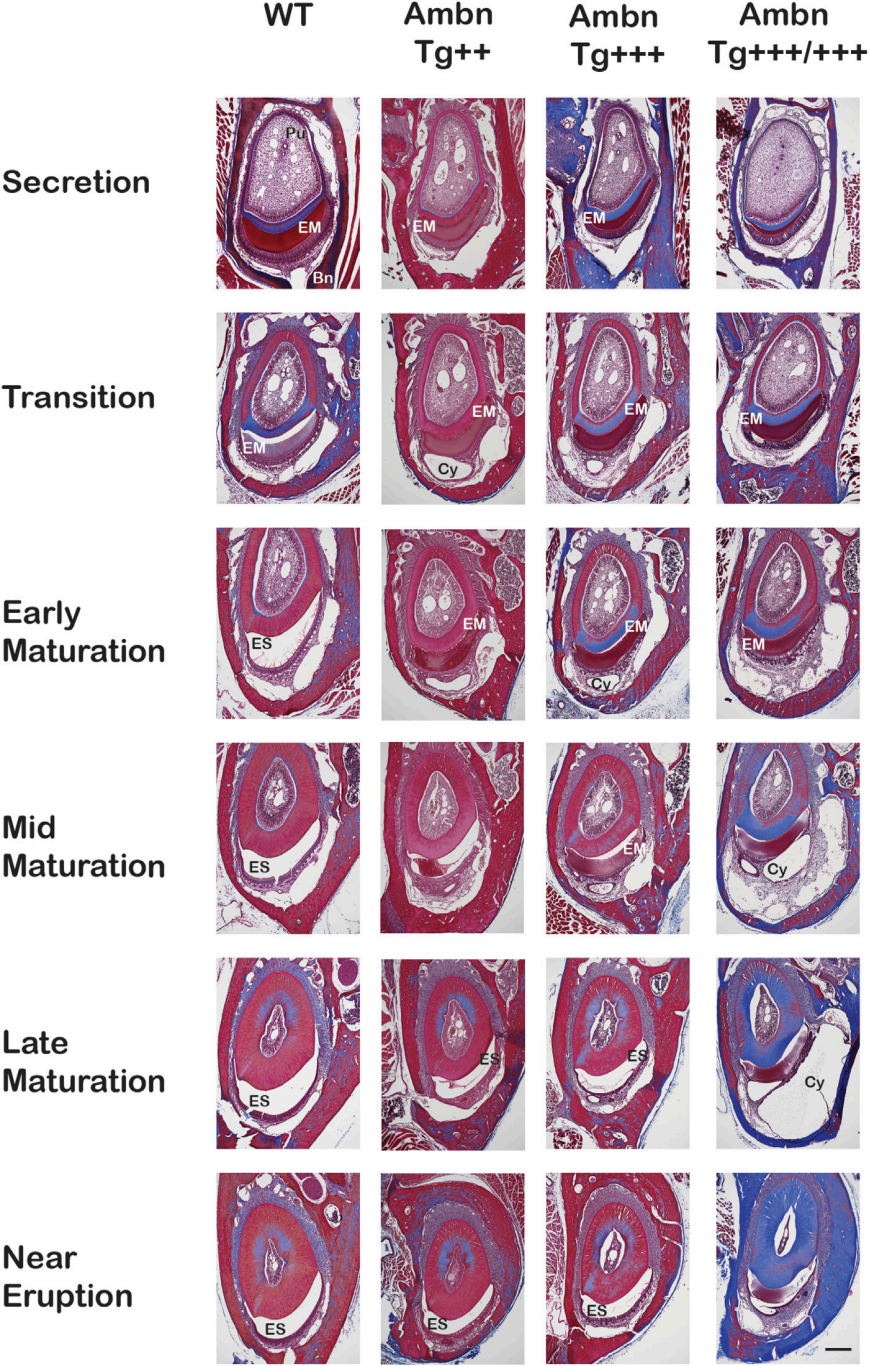
Transverse sections of mandibular incisors stained with Masson’s trichrome. Hemi-mandibles were transversely sectioned at six defined levels from the apical loop to the eruption point and stained with Masson’s trichrome. At the secretory stage, the enamel matrix (EM) was deposited. In wild-type (WT) animals, the matrix diminished at the transition stage and was almost completely absent from the maturation stage, represented by the enamel space (ES) in decalcified samples. In *Ambn Tg* animals, the matrix removal was delayed by two levels (mid-maturation) in *Ambn Tg* ^*+++*^ and near eruption in *Ambn Tg* ^*+++/+++*^. The enamel organ expanded greatly in *Ambn Tg* ^*+++/+++*^ incisors in middle and late maturation stages, harboring cysts (Cy). EM, enamel matrix; ES, enamel space; Bn, bone; Pu, pulp; Cy, cyst. Scale bar: 200 μm.

**FIGURE 6:**
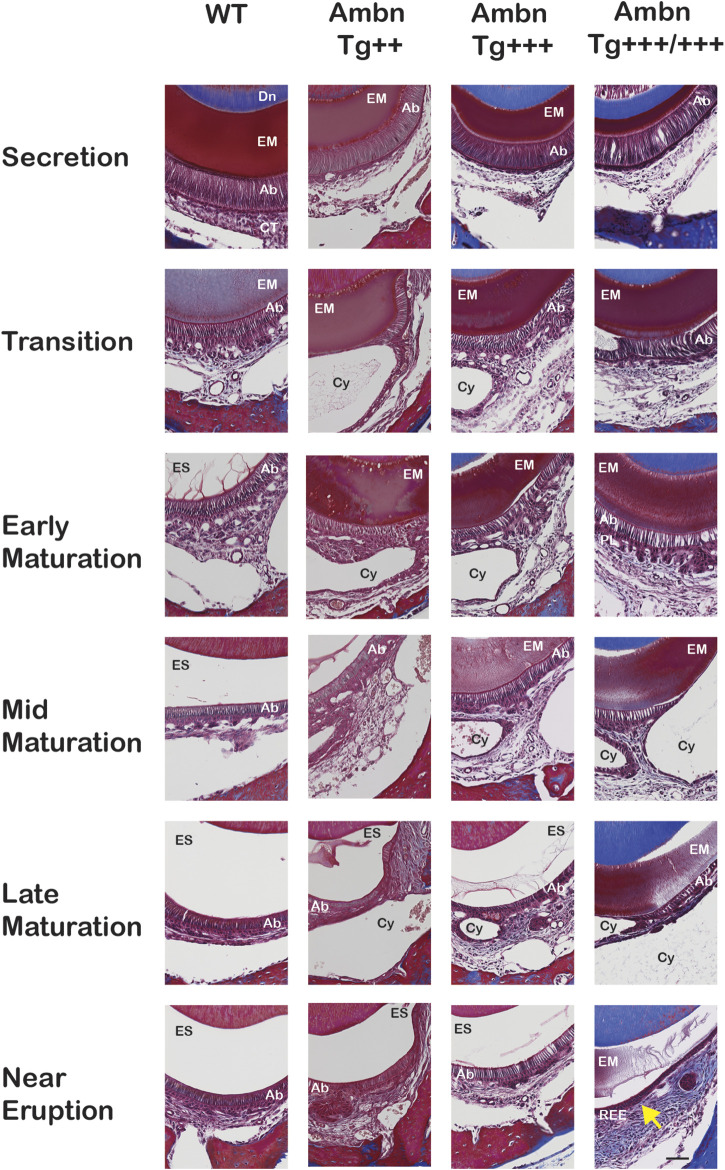
Delayed removal of the enamel matrix and early transition into the reduced enamel epithelium. In wild-type (WT), the dental epithelium differentiated into a stratified enamel organ to support the secretion of enamel proteins by polarized ameloblasts with Tomes’ process. In *Ambn Tg* animals, ameloblasts were similar in height but less connected to each other. The connective tissue surrounding the enamel organ was less developed. In the transition stage ameloblasts, a papillary layer (PL) was developed at the base of ameloblasts but was reduced in height in *Ambn Tg* ^*+++/+++*^. In the early maturation stage, the papillary layer demonstrated irregularities in organization, and cysts (Cy) were noted in the maturation stage. In the late maturation stage and near eruption, the enamel organ of *Ambn Tg* ^*+++/+++*^ collapsed into a cell layer of flattened morphology (yellow arrow), resembling the reduced enamel epithelium (REE). Ab, ameloblast; EM, enamel matrix; ES, enamel space; PL, papillary layer; Dn, dentin; CT, connective tissue; Cy, cyst. Scale bar: 50 μm.

The progression of enamel matrix deposition and removal was followed in incisors from secretory to early, middle, and late maturation stages (Figures 4A; Figure 5; Figure 6). Enamel matrix is secreted and deposited in wild-type animals in the secretory stage and intensely degraded in the early maturation stage, leading to the resorption of peptides from the matrix through ameloblasts. When *Ambn* was overexpressed, the enamel matrix was not timely removed. Greater quantities of enamel proteins stayed longer in the enamel, starting at the secretory stage of *Ambn Tg* ^*+++*^ and *Ambn Tg* ^*+++/+++*^ (Figures 4A; Figures 5, 6). Residuals of the enamel matrix were found adjacent to areas of detached ameloblasts in the middle and late maturation stages in *Ambn Tg* ^*+++*^ animals (Figures 4A, B; Figures 5, 6). Large protein quantities were observed throughout the thickness of the enamel layer in *Ambn Tg* ^*+++/+++*^ up to the brink of eruption (Figures 4A, B; Figures 5, 6). The retained enamel protein matrix coincided with cysts in the enamel organ that occupied the enamel organ up to tooth eruption in *Ambn Tg* ^*+++/+++*^ animals (Figures 4A, B; Figures 5, 6). Cysts that were in close proximity to the enamel surface demonstrated flattened, detached ameloblasts and a subjacent retained protein matrix (Figures 4A, B). In contrast, ameloblasts reduced in height but directly attached to the enamel exhibited less protein content in the matrix (Figures 4A, B). At the late maturation stage, the layers of the enamel organ merged and adopted a flat appearance, resembling the reduced enamel epithelium earlier than in wild-type animals (Figure 6). Ambn overexpression induced morphological changes of ameloblasts and the papillary layer, visible as detachment and cysts, both linked to enamel protein retention.

### Ameloblastin localized at the mineralization front and dentino-enamel junction

**FIGURE 7:**
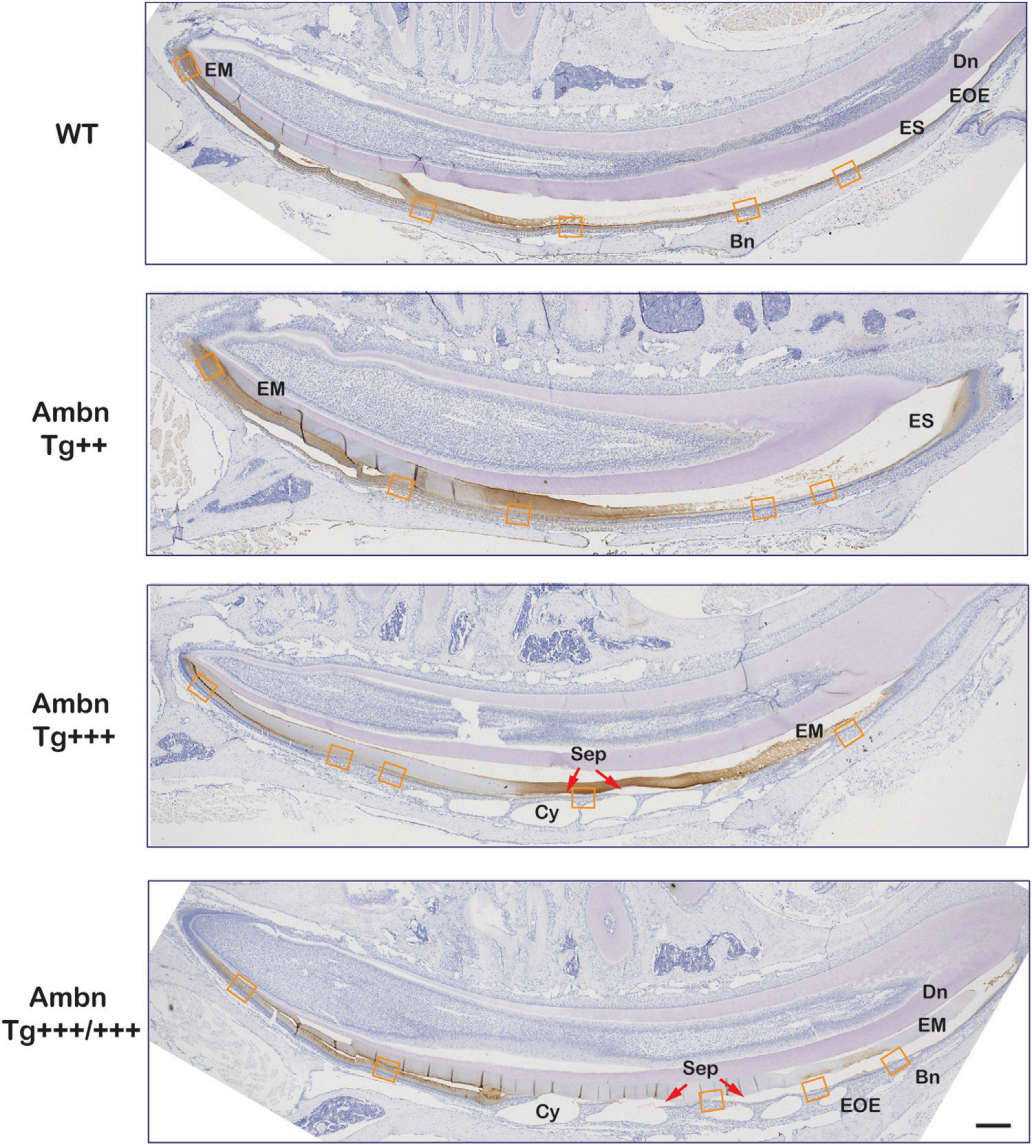
Secretory and maturation stage immunolocalization of Ambn in incisors. In sagittally sectioned mandibular incisors, enamel formation advanced from secretory (left) to maturation and eruption (right). In the wild-type (WT), the enamel matrix and the enamel organ epithelium (EOE) demonstrated immunolocalization of Ambn during secretory and maturation stages. In the middle and late maturation stages, the reduction of the bulk of the enamel matrix (EM) coincided with the modulation of the immunosignal strength between stronger and weaker regions. In *Ambn Tg* ^*++*^ and *Ambn Tg* ^*+++*^ animals, the enamel matrix was retained into the middle and late maturation stages. *Ambn Tg* ^*+++*^ animals exhibited sequentially staggered cysts in EOE. Ameloblasts closest to the zenith of cysts (Cy) were detached (red arrow). In *Ambn Tg* ^*+++/+++*^ animals, ameloblasts with features of transition-stage ameloblasts were found in the early maturation stage. Modulation of Ambn signals was absent, and the enamel matrix was retained in the late maturation stage. The orange frames correspond to the magnified images shown in Figure 8. Dn, dentin; EM, enamel matrix; ES, enamel space; EOE, enamel organ epithelium; Bn, bone; Cy, cyst; Sep, separation; red arrows point at detached ameloblasts. Scale bar: 200 μm.

**FIGURE 8:**
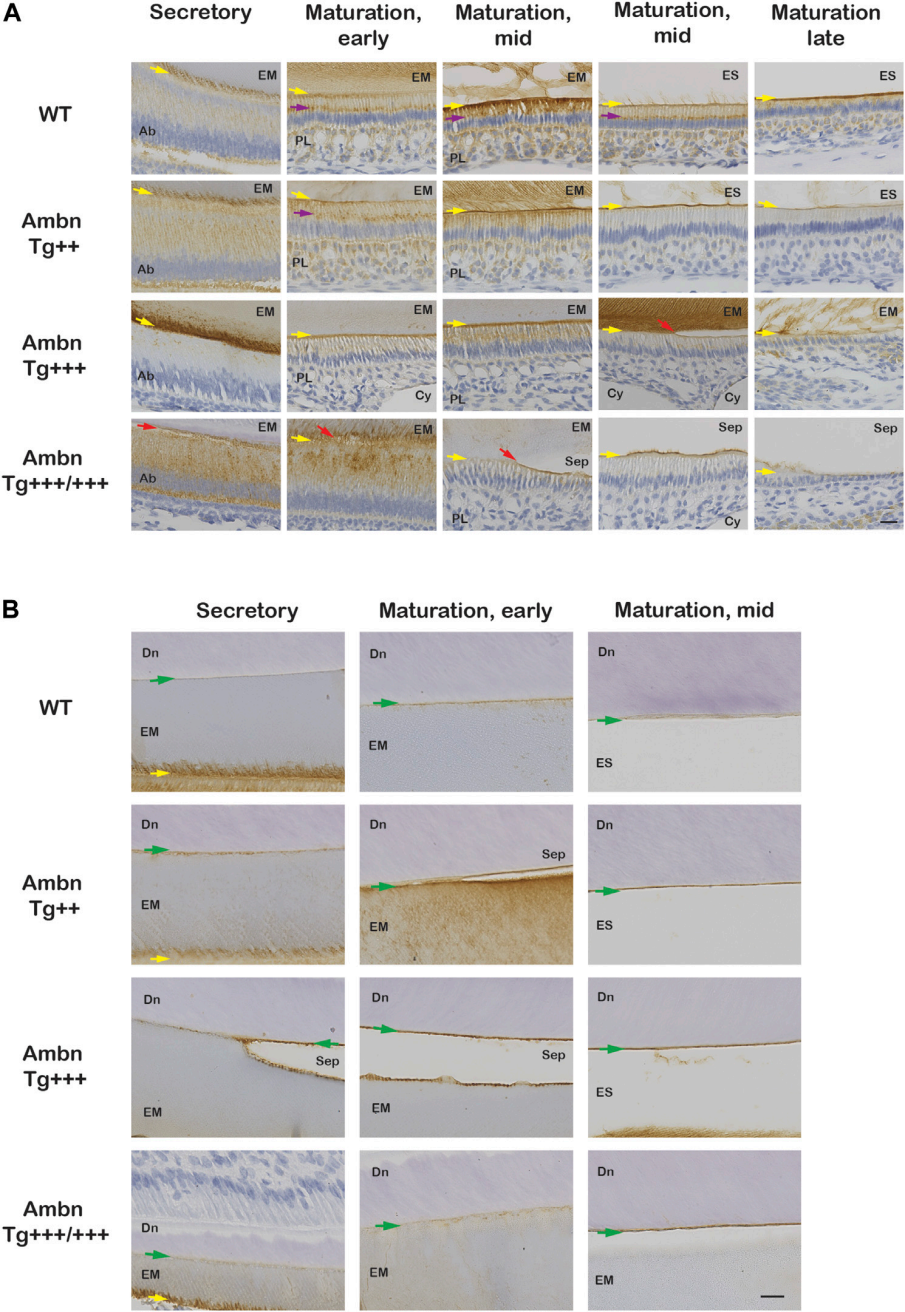
Ambn immunolocalization at the ameloblast–enamel interface and the dentino-enamel junction with overexpressed *Ambn*. The secretory and maturation stages of enamel formation were analyzed for Ambn localization in mandibular incisors of 7-week-old animals. **(A)** At the secretory stage, strong Ambn localization consistent with overexpression was apparent in the cytoplasm (*Ambn Tg* ^*++*^) and at the mineralization front (yellow arrow; *Ambn Tg* ^*+++*^). In *Ambn Tg* ^*+++/+++*^ expression, signals surrounded the perinuclear region in foci, overlapping with the region of the endoplasmic reticulum and secretory vesicles. Ameloblasts detached easily from the enamel matrix (EM) (red arrow). In the maturation stages, Ambn localization in the Golgi apparatus (purple arrow), in the basement membrane, and in papillary layer (PL) cells was reduced compared to wild-type (WT) when Ambn was overexpressed. In the late maturation stage, ameloblasts reduced in height earlier when Ambn was overexpressed (*Ambn Tg* ^*+++/+++*^). Scale bar: 20 μm. **(B)** Ambn was found in WT at the dentino-enamel junction (DEJ) (green arrow) and accumulated more when Ambn was overexpressed. Separations (Sep) at the DEJ were a common finding in *Ambn Tg* ^*++*^, *Ambn Tg* ^*+++*^, and *Ambn Tg* ^*+++/+++*^, suggesting biological rather than traumatic weakness or separation. Ab, ameloblast; EM, enamel matrix; PL, papillary layer; ES, enamel space; Sep, separation; Cy, cyst; Dn, dentin; the yellow arrows indicate the interface of the ameloblast plasma membrane and enamel matrix/enamel space, the red arrow points represent detached ameloblasts, and the green arrows are directed at the dentino-enamel junction (DEJ). Scale bar: 20 μm.

**FIGURE 9:**
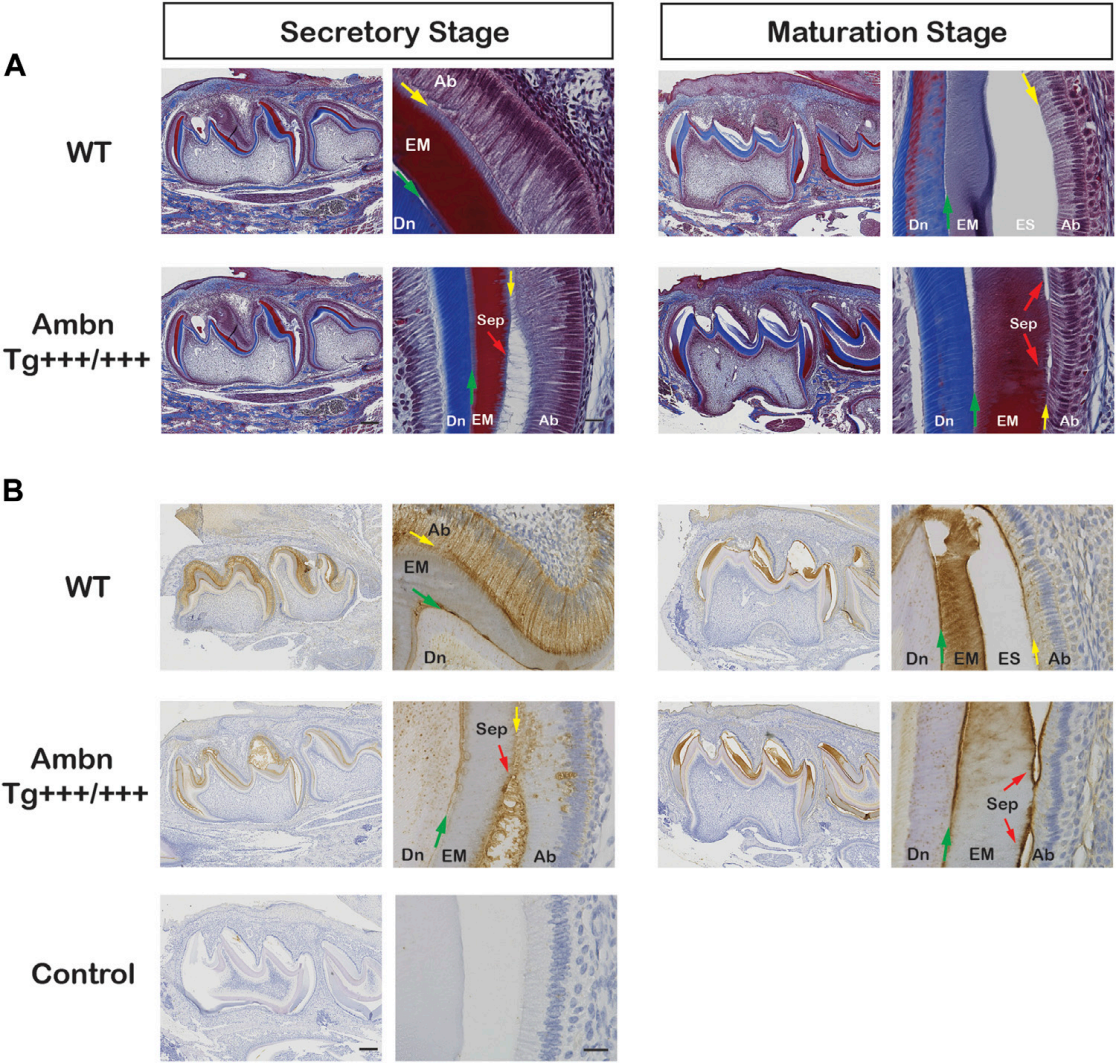
Ambn overexpression in molars during enamel formation. The secretory and maturation stages of enamel formation were analyzed in mandibular first molars at postnatal days 5 and 11. **(A)** Masson’s trichrome staining highlights the separation (Sep) of ameloblasts from the enamel matrix in secretory and maturation stages (red arrow) in the presence of overexpressed Ambn. **(B)** Ambn localization was found in ameloblasts (Ab), enamel matrix (EM), and at the dentino-enamel junction (DEJ, green arrow) in the wild-type (WT). With *Ambn Tg* ^*+++/+++*^ expression, Ambn localization reduced in ameloblasts except for large perinuclear vesicles, and Ambn was increased at the DEJ. Ameloblasts in secretory and maturation stages separated from the enamel matrix in the presence of overexpressed Ambn (red arrow). Ab, ameloblast; EM, enamel matrix; ES, enamel space; Dn, dentin; Sep, separation; the red arrows indicate detached ameloblasts, the yellow arrows indicate the interface of the ameloblast plasma membrane and enamel matrix/enamel space, the green arrows are directed at the dentino-enamel junction (DEJ). Left scale bar for low magnification, 200 μm; right scale bar for high magnification, 20 μm.

Ambn was present in all stages of enamel formation, starting in secretory, and maturation ameloblasts of wild-type incisors and molars (Figures 7–9; Supplementary Figure S1). In the secretory stage, Ambn resided throughout the entire cell body in the supra- and infra-nuclear regions of polarized ameloblasts in the wild-type and *Ambn* overexpressers. Signals were particularly strong at the Tomes’ process and mineralization front of *Ambn Tg* ^*++*^ and *Tg* ^*+++*^ (Figure 8A; Figure 11A; Supplementary Figure S1). At the mineralization front, the Ambn signal changed from angled and fringe-like appearance relative to the plasma membrane in wild-type to a dense band-like appearance in *Ambn Tg* ^*+++*^ (Figure 8A; Supplementary Figure S1), suggesting that secreted Ambn accumulated and possibly was not adequately resorbed. In *Ambn Tg* ^*+++/+++*^, the signals at the mineralization front were reduced and disorganized, and ameloblasts detached easily at the mineralization front related to the altered shape and reduced height of the Tomes’ process. Accumulation of Ambn in discrete round granules within the cytoplasm was increased in *Ambn Tg* ^*+++/+++*^ *,* representing secretory vesicles (Figure 8A; Figure 9B). Tall, polarized ameloblasts were found beyond the usual length of the secretory stage.

Wild-type ameloblasts of early and mid-maturation stages demonstrated immunosignals in the basement membrane deposited on the enamel surface in alternating stretches of stronger and weaker signals (Figure 7). Signals in the sub-nuclear region in wild-type represented endocytosed Ambn in the Golgi apparatus (Figure 8A; Figure 9B). The papillary layer presented with immunosignals evenly distributed across the cell body (Figure 8A). When *Ambn* was overexpressed, immunosignals gradually reduced in the Golgi apparatus, papillary layer, and basement membrane of *Ambn Tg* ^*++*^, *Ambn Tg* ^*+++*^, and *Ambn Tg* ^*+++/+++*^, respectively. The reduction of the Golgi signal suggests that fewer Ambn peptides were endocytosed by ameloblasts. In the highest *Ambn* expressers, ameloblasts drastically reduced in height and transitioned into the reduced enamel epithelium earlier than in wild-type animals. The matrix was retained when ameloblasts separated from the enamel surface, in proximity to the zenith of cysts formed within the enamel organ of *Ambn Tg* ^*+++*^ and *Tg* ^*+++/+++*^ (Figures 9A, B). The alteration of the ameloblast morphology, basement membrane, and/or the mechanical pressure of cysts may contribute to the detachment of ameloblasts from the enamel surface.

In addition to the ameloblast mineralization front, Ambn localized to the DEJ in the increasing quantity from wild-type to the highest *Ambn* expressers (Figure 8B; Figure 9B; Figure 11A). The enamel matrix separated easily from the dentin when Ambn was overexpressed (Tg^++^, Tg^+++^, and Tg^+++/+++^), possibly due to weakening of the junction or biological separation. The presence of too much Ambn between ameloblasts and mineralization front in the secretory stage, basement membrane, and enamel surface, and dentin and enamel weakened the transition and resulted in protein retention.

### Dysregulation of enamel proteins

**FIGURE 10:**
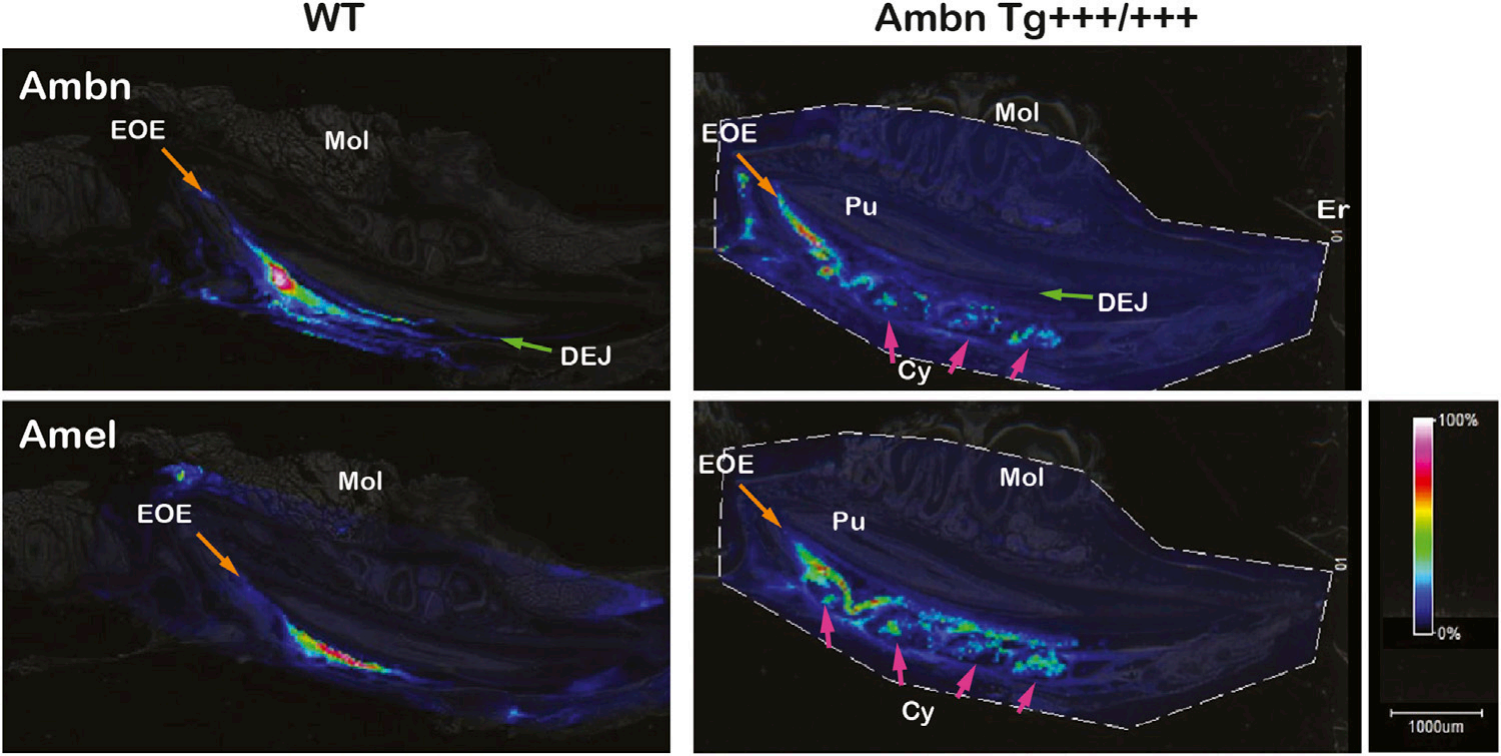
Identification and localization of enamel proteins in incisors by mass spectrometry imaging. Ameloblastin (Ambn) and amelogenin (Amel) peptides were identified in secretory-stage enamel of wild-type incisors. Ambn localized to the dentino-enamel junction (DEJ; green arrow) and the enamel organ epithelium (EOE; orange arrow). In *Ambn Tg +++/+++*, Ambn and Amel signals persisted longer in the maturation stage. Cysts (Cys) within the enamel organ epithelium contained Ambn and Amel peptides (pink arrows). Er, eruption point; EOE, enamel organ epithelium; Mol, molar; Pu, pulp; DEJ, dentino-enamel junction; Cy, cyst.

Mass spectrometry imaging directly identifies peptides in tissues without the use of antibodies. In wild-type animals, amelogenin (Amel) was identified at m/z 983 corresponding to WYQSMIR, and Ambn at m/z 1744 corresponding to QLGSLQGLNALSQYSR. In wild-type animals, Amel and Ambn peptides localized strongly to the secretory stage. Ambn peptides were detected earlier in development than Amel peptides. Although Amel was confined to bulk enamel, Ambn was found in bulk enamel and in enamel close to the DEJ and the enamel organ (Figure 10). In *Ambn Tg* ^*+++/+++*^ mandibles, Ambn and Amel signals were found in the secretory stage and were retained longer into the maturation stage. Amel and Ambn signals were also found in the lumen of cysts residing within the enamel organ.

### Ameloblastin secretion and endocytosis

**FIGURE 11:**
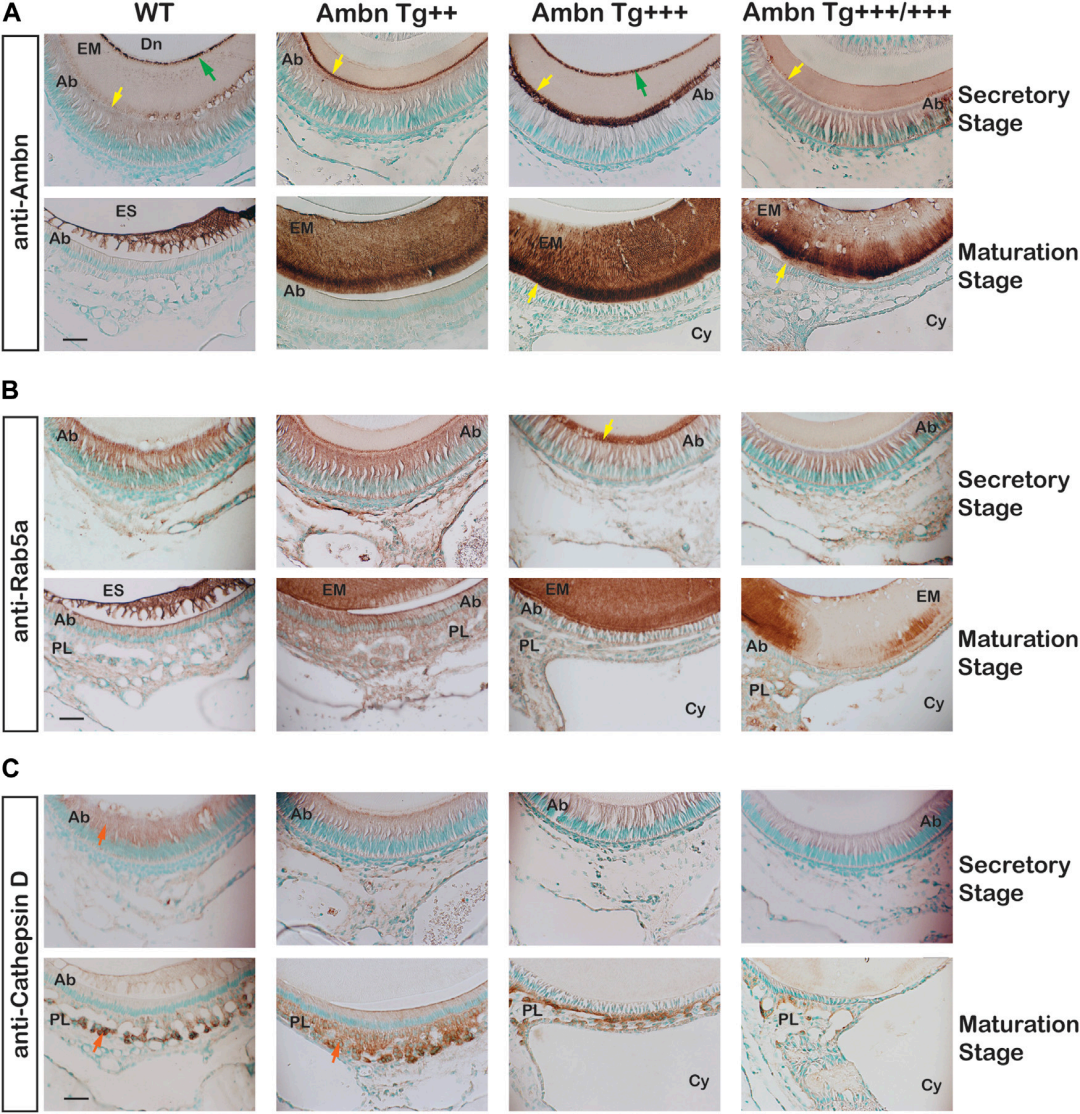
Endocytosis by ameloblasts in the presence of Ambn overexpression. The effects of Ambn quantity on secretion and endocytosis were analyzed by immunolocalization on transversely sectioned mandibular incisors. **(A)** In *Ambn Tg+++,* strong localization was noticed at the mineralization front (yellow arrowheads) and the dentino-enamel junction (green arrow). In *Ambn Tg* ^*+++/+++*^ *,* the immunosignals were weaker than in *Ambn Tg* ^*+++*^. At the maturation stage, the enamel matrix was retained. **(B)** Rab5a immunosignals marking endosomes were uniformly found in ameloblasts of secretory and maturation stages in WT. The Rab5a response to Ambn overexpression was distinct. Rab5a immunosignals increased in the ameloblast (Ab) cytoplasm of *Ambn Tg* ^*++*^ and at the mineralization front of *Ambn Tg* ^*+++*^ (yellow arrow) but reduced in the cytoplasm of *Ambn Tg* ^*+++*^ and *Tg* ^*+++/+++*^. **(C)** Cathepsin D indicated lysosomal degradation in the secretory and maturation stages of ameloblasts and the papillary layer (orange arrow). In *Ambn Tg* ^*++*^, signals were increased compared to WT and decreased in *Ambn Tg* ^*+++*^ and *Tg* ^*+++/+++*^. Ab, ameloblast; EM, enamel matrix; ES, enamel space; Dn, dentin; PL, papillary layer; Cy, cyst; the yellow arrows indicate the interface of the ameloblast plasma membrane and enamel matrix, and the green arrows are directed at the dentino-enamel junction (DEJ). Scale bar: 50 μm.

In the secretory stage, Ambn highlighted localizations at the mineralization front close to Tomes’ process of Ambn Tg^++^ and Ambn Tg^+++^ and the DEJ of Ambn Tg^+++^ (Figure 11A). In the maturation stage, Ambn was retained within the matrix, suggesting that endocytosis lacked the efficiency to remove extracellular peptides. Markers for endocytosis are Rab GTPases located in endosomes that shuttle extracellular peptides to lysosomes (Pham et al., 2017). The ameloblast activity related to the removal of enamel proteins through endosomes was represented by early endosomes expressing Rab5a in secretory and maturation stages. Rab5a signals were evenly dispersed throughout the cell body in wild-type animals (Figure 11B). Signals became stronger in the cytoplasm of secretory and maturation stage ameloblasts in *Ambn Tg* ^*++*^ mice. In the *Ambn Tg* ^*+++*^ genotype, Rab5a was strongly present at the mineralization front but reduced in the ameloblast cytoplasm (*Tg* ^*+++*^ and *Tg* ^*+++/+++*^) at secretory and maturation stages. The lysosomal serine protease cathepsin D is generally found in lysosomes to degrade proteins (Zaidi et al., 2008) and is expressed by the enamel organ epithelium (Tye et al., 2009). Cathepsin D localized to the cytoplasm of secretory and maturation stage ameloblasts and most strongly to cells of the papillary layer (Figure 11C). Papillary layer cells contain tubular lysosomes known to degrade endocytosed proteins (Salama et al., 1990). In *Ambn Tg* ^*++*^, the papillary layer around capillaries was expanded, coinciding with strong immunoreactivity for cathepsin D (Figure 11C). However, in the highest *Ambn* expression, cathepsin D expression decreased (Figure 11C). In *Ambn Tg* ^*++*^ mice, the increase in Ambn was largely compensated by increased endocytosis and protein degradation. When the *Ambn* expression increased further (*Tg* ^*+++*^ and *Tg* ^*+++/+++*^), compensation for these activities could not be sustained, and Ambn was no longer efficiently endocytosed or degraded and accumulated at the mineralization front (Figure 11A). Reduced Rab5 and cathepsin D expressions were consistent with decreased protein endocytosis and decreased lysosomal degradation, leading to unusual protein retention in the enamel matrix.

## Discussion

The under- or overexpression of *Ambn* in six mouse lines for the first time resulted in a spectrum of hypoplastic and hypomineralized enamel and disruption of the morphology and function of ameloblasts and the enamel organ. Hypoplastic amelogenesis imperfecta with thin enamel, irregular mineral foci, and ectopic minerals is caused by the underexpression of *Ambn* (Fukumoto et al., 2004; Liang et al., 2019). When overexpressed in mice, Ambn lacks efficiency undergoing cleavage, cell attachment, resorption, and increases in mineral content, thus resulting in hypomineralized opacities surrounded by the unaffected enamel. The increase in the Ambn concentration in four transgenic mouse lines displays an increase of lesions in severity and extent. In conjunction with ameloblast pathophysiology, the initiation and progression of opaque enamel hypomineralization was traced. Our Ambn model is the first to report demarcated, hypomineralized opacities in the enamel. In a previous mouse model overexpressing Ambn, opaque hypomineralized enamel was observed, albeit without demarcations (Paine et al., 2003). The model by Paine may overexpress Ambn at a lower concentration, differing from our model in the design of the promoter, signal peptide, and 3′UTR (Chun et al., 2010).

**FIGURE 12:**
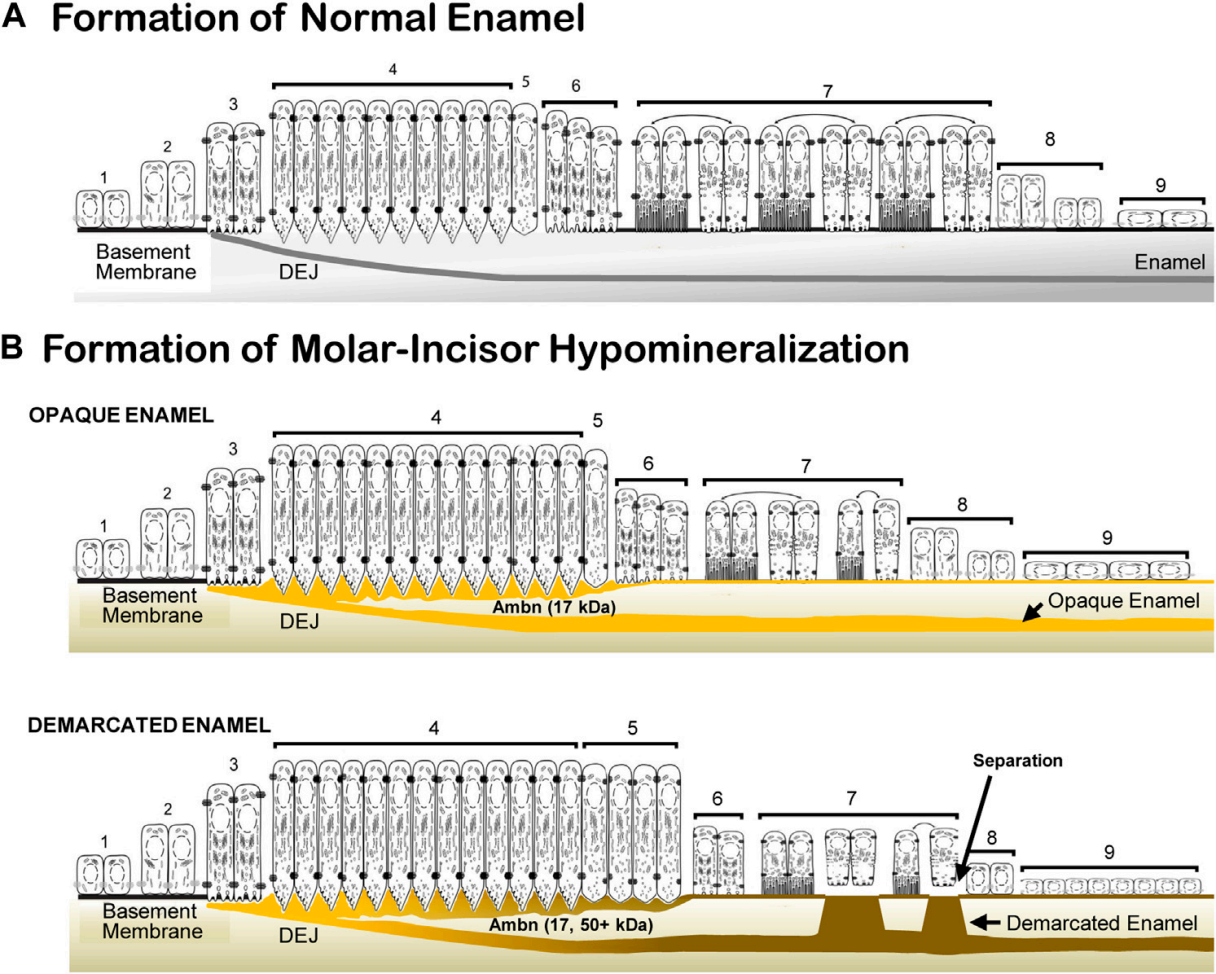
Schematic representation of normal enamel formation and molar–incisor hypomineralization. **(A)** Enamel forms in sequential stages. Undifferentiated inner epithelial cells are attached to a basement membrane (1, 2). Lengthened and polarized pre-ameloblasts form processes reaching through the degraded basement membrane to create the dentino-enamel junction (DEJ) (3). Ameloblasts with Tomes’ processes secrete enamel proteins that control the initiation, organization, and extension of enamel rods (4). During the post-secretory transition (5), ameloblasts reduce in length, the Tomes’ process flattens for early maturation stage ameloblasts (6), and a basement membrane is deposited, to which modulating ameloblasts attach with ruffled and smooth plasma membranes (7). Post-maturation ameloblasts are reduced in height (8) and become a reduced enamel epithelium (9) until the tooth erupts into the oral cavity. **(B)** In molar–incisor hypomineralization, lesions appear opaque when the inner enamel layer is affected, and demarcated when the entire enamel layer is affected. In opaque lesions, the initiation of amelogenesis occurs, as in normal formation (1, 2, and 3). The duration of the secretory stage is longer than in normal enamel formation (4). The overexpressed Ambn (yellow) accumulates as a 17-kDa cleavage product in the inner enamel close to the DEJ and at the mineralization front. Post-secretory ameloblasts (5, 6) become condensed maturation stage ameloblasts and attach to a thin basement membrane (7). In demarcated lesions, in addition to the 17-kDa Ambn cleavage product, Ambn species of higher molecular weight accumulate in the inner enamel and mineralization front. The secretory and transition stages are prolonged (4, 5). Maturation stage ameloblasts are decreased in height and alternate between attached and detached states directly associated with the retention or resorption of proteins (7). Post-maturation (8) and reduced enamel epithelium (9) are initiated earlier compared to normal enamel formation (adapted and modified from Hu et al. 2007).

Enamel formation is a sequential process of morphologically and functionally defined stages of pre-ameloblast, secretion, transition, maturation, and the reduced enamel epithelium (Hu et al., 2007). In the *Ambn* overexpression mouse model, the morphological and functional deviations caused enamel hypomineralization. The deposition of increasing quantities of Ambn at the DEJ and mineralization front did not cause loss of cell polarity. Instead, a series of events occurred: impaired cleavage of full-length Ambn, impaired endocytosis, prolonged secretory and transition stages, thin basement membrane, detached ameloblasts, and premature transition into the reduced enamel epithelium, resulting in an abbreviated maturation stage and impaired mineralization (Figure 12). The approach of increasing Ambn concentrations allowed the comparison of the development of lesions and the incremental changes in the enamel organ epithelium. The timing for advancing to the next stage, critical for amelogenesis, relies on feedback loops to continuously monitor the state of amelogenesis and adjust the ameloblast function. This step-wise process has built-in checkpoints to allow tight regulation for precise timing of the stages. Too much of Ambn induced changes in the ameloblast program, resulting in opaque, demarcated enamel hypomineralization.

### Etiology of MIH recapitulated in animal models

The need for understanding the precise pathophysiology of MIH is substantial since it affects children globally and impacts their oral health, growth, and development (Lopes et al., 2021; Lygidakis et al., 2022). The etiological factors of MIH are linked to prenatal maternal illness, perinatal hypoxia, and postnatal childhood illness, suggesting genetic and epigenetic complexities (Garot et al., 2022). Ameloblasts forming permanent teeth could be susceptible to stressors in the form of perinatal and postnatal illnesses and birth. Systemic and environmental factors may exceed the capacity of ameloblasts to mitigate stress and result in dysfunction manifesting as MIH.

With clinical studies limited to erupted, exfoliated, and extracted teeth and precluding studies of the enamel organ, which is lost to apoptosis in the transition to tooth eruption (Shibata et al., 1995), animal models provide access to pre-eruptive stages of tooth development. Hypomineralized, opaque enamel has been produced in animal models caused by bisphenol A (BPA) and amoxicillin, in which dysregulation of enamel proteins was found (Duman et al., 2022). Rats exposed to BPA exhibited enamel hypomineralization of the incisal tips (Jedeon et al., 2013). However, demarcated opacities have been produced in a few animal models. Sheep displayed demarcated opacities in enamel after infection with parasites through systemic effects of the gastrointestinal metabolism (Suckling et al., 1983). The enamel retained an organic matrix, and the organization of the enamel organ was disturbed by cysts, shorter ameloblasts, and ameloblast separation from the matrix (Suckling et al., 1986). The demarcated opacities were only found in the cervical region but not in coronal areas, supporting that demarcations originated in the secretory stage. Similarity of the quality of demarcation in sheep and Ambn overexpressing mice suggests a common pathophysiology. Although the genetic and epigenetic mechanisms leading to an upregulation of the *Ambn* gene are unknown, a single-nucleotide polymorphism (SNP) of the *AMBN* gene was associated with MIH (Jeremias et al., 2016). Furthermore, the *MMP20* gene was recently identified as a locus for MIH (Hočevar et al., 2020). SNPs of *Ambn* and *MMP20* could affect the turnover rate of enamel proteins. Furthermore, ameloblastin protein was identified in the enamel of extracted teeth affected by MIH by mass spectrometry (Farah et al., 2010). This finding is noteworthy because secreted Ambn is efficiently cleaved by Mmp20 (Chun et al., 2010). Ambn is detected at the mineralization front of the enamel surface but does not accumulate in enamel layers (Hu et al., 1997).

### Onset and location of enamel hypomineralization related to Ambn overexpression

Studies with extracted human teeth affected by MIH demonstrated that lesions start at the DEJ and are confined to the inner enamel in mild cases while surrounded by sound outer enamel (Fearne et al., 2004; Fagrell et al., 2013). In more severe cases, the lesion extends from the DEJ toward the enamel surface, following incremental lines (Farah et al., 2010; Fagrell et al., 2013). The onset of MIH was modeled by *Ambn* overexpression in four increasing Ambn concentrations that produced a range of hypomineralization severities. In this mouse model, full-length Ambn was overexpressed in the secretory stage of enamel formation. Similar to wild-type, Ambn localized to the mineralization front and the DEJ in the secretory stage and to the basement membrane in the maturation stage. The lowest overexpression of *Ambn Tg* ^*+*^ did not produce demarcation, discoloration, or hypomineralization of the enamel. Mild hypomineralization was displayed in *Ambn Tg* ^*++*^ animals with reduced translucency that started at the secretory stage with Ambn deposited at the DEJ, and hypomineralized lesions were confined to the inner enamel. The delay in resorption of the enamel matrix suggests an underlying defect in the ameloblasts initiated in the secretory stage. Demarcated opacities were apparent in *Ambn Tg* ^*+++*^ animals spanning the width of the enamel layer from the DEJ to the enamel surface. Lesions were associated with retained enamel matrix, a reduced basement membrane, and detached ameloblasts. In the most severe lesions of *Ambn Tg* ^*+++/+++*^, the hypomineralized enamel was expanded, and demarcations were blurred. The enamel layer was thinner in mouse molars originating from the secretory stage, as observed in MIH-affected teeth (Patel et al., 2019; Bittencourt and Cesario, 2022). Ambn accumulated at the DEJ in transgenic mice, initiating hypomineralized lesions and opaque color. A high concentration of Ambn increased the defect size and reduced the thickness of the enamel layer (Figure 12).

### Ameloblastin in the innermost enamel, cleavage by Mmp20, and delamination from dentin

The *Ambn* overexpressing animals deposited Ambn along the DEJ, on the side of the innermost enamel. The Ambn quantities in *Tg* ^*+*^ and *Tg* ^*++*^ mice in the innermost enamel resulted in hypomineralization without mechanical failures. However, *Ambn Tg* ^*+++*^ and *Tg* ^*+++/+++*^ enamel had hypomineralized lesions spanning the entire thickness of the enamel, fractures of load-bearing sites, and delaminated enamel at the incisal tip. The innermost enamel adjacent to the DEJ is the location that contains the most proteins and the least mineral of erupted teeth (Robinson et al., 1971; Dusevich et al., 2012). The sheath surrounding enamel rods contains organic material, including Ambn (Uchida et al., 1991; Hu et al., 1997; Dusevich et al., 2012). Analysis by nano-LC-mass spectrometry identified Ambn from the DEJ (Jágr et al., 2018). Organic material at the DEJ adds elasticity at the junction to absorb forces and prevent catastrophic failures during mastication (Bechtl et al., 2010; McGuire et al., 2014). However, when the DEJ contains more proteins than the wild-type, it fails to withstand masticatory forces and delaminates in *Ambn* overexpressers. Similarly, in MIH patients, affected teeth commonly experience post-eruptive breakdown upon occlusal load.

The insufficient cleavage of enamel proteins by proteinases causes enamel delamination from the underlying dentin (Caterina et al., 2002; Simmer et al., 2012). Mmp20 is expressed by odontoblasts and secretory stage ameloblasts (Begue-Kirn et al., 1998) and cleaves proteins necessary for proper mineralization at the junction of enamel and dentin (Nagano et al., 2009; Chun et al., 2010). In the absence of Mmp20, the enamel separates at the DEJ in *Mmp20* null mice (Simmer et al., 2012), and in the absence of Klk4, the enamel fractures within the initial enamel (Simmer et al., 2011). Mmp20 recognizes a conserved site after Arg170, releasing a 17-kDa Ambn as an initial cleavage product (Chun et al., 2010). In *Ambn Tg* ^*+++*^ and *Tg* ^*+++/+++*^ mice, in addition to the N-terminal 17 kDa, an N-terminal 50 kDa Ambn cleavage product appeared unexpectedly, suggesting that the release of the 17-kDa cleavage product was incomplete. Similarly, in *Mmp20* null mice, the Ambn 17-kDa fragment was not generated, but a 50-kDa Ambn N-terminal fragment was found instead (Yamakoshi et al., 2011). Secondary cleavage sites by Klk4 recognize Arg319 in pig Ambn and predict a 48-kDa fragment (Yamakoshi et al., 2001). The homologous, conserved Arg325 of mouse Ambn releases a fragment containing six additional residues that account for the difference in 2 kDa. In the secretory stage, full-length Ambn is efficiently cleaved by Mmp20 at the initial cleavage site after Arg170 (Chun et al., 2010). The appearance of larger N-terminal fragments of 50 kDa and higher is unexpected in *Ambn Tg* ^*+++*^ and *Tg* ^*+++/+++*^, suggesting that the enzymatic efficiency of Mmp20 could have been overwhelmed by an excess of Ambn, rendering the cleavage site intact in some molecules. Maturation stage Klk4 may have cleaved full-length Ambn instead of Mmp20, generating the higher-molecular weight Ambn fragments from secondary cleavage sites.

The localization and cleavages of the C-terminal Ambn in transgenic Ambn overexpressing animals are unknown. C-terminal Ambn is characterized by acidic pI, and the intrinsic disordered propensity is proposed to facilitate crystal growth (Wald, 2011; EJOS). The C-terminus is cleaved by Mmp20 into small peptides consisting of 53, 76, and 95 residues in the pig enamel (Yamakoshi et al., 2001; Chun et al., 2010). These peptides were observed close to the mineralization front and not within the bulk enamel, suggesting a role in guiding crystals to elongate enamel ribbons (Uchida et al., 1998).

### Timing of enamel formation, responsiveness of the enamel organ, and endocytosis

The sequential progression of enamel formation is tightly regulated through feedback between the matrix and ameloblasts. The mineralization front is positioned to facilitate the elongation of enamel ribbons, the deposition of ions, and the relay of feedback. Ambn localizes to the mineralization front in proximity to the ameloblast membrane (Hu et al., 1997; Uchida et al., 1998). When *Ambn* was overexpressed (*Ambn Tg* ^*++*^, *Tg* ^*+++*^ *,* and *Tg* ^*+++/+++*^), the secretory stage was prolonged, and Ambn strongly localized to the Tomes’ process and mineralization front in the secretory stage. The accumulations may be the result of impaired processing and impaired resorption of Ambn peptides. In *Ambn Tg* ^*+++/+++*^ animals, the cytoplasm and mineralization front were less immunopositive for Ambn signals. The transition stage marked by blunted Tomes’ processes was prolonged, compared to the wild-type. Ambn localized evenly in the cytoplasm at the secretory stage of *Ambn Tg* ^*++*^, but in *Ambn Tg* ^*+++*^ and *Tg* ^*+++/+++*^ mice, Ambn accumulated in visibly large vesicles. The prominent vesicles could represent secretory vesicles that are en route to be released at the plasma membrane or endocytic vesicles containing cleaved Ambn peptides destined for lysosomal degradation but facing difficulties in releasing their content. Secretory ameloblasts contain large vesicles with Ambn (Zalzal et al., 2008; Pandya et al., 2017). The changes signify responses of a finely tuned and highly responsive regulatory cell program. As an initial response to Ambn overexpression (*Tg* ^*++*^), endocytic and lysosomal activities increased to deal with the increased matrix substrate. However, the continued increase in Ambn (*Tg* ^*+++*^ and ^*+++/+++*^) led to a reduced height of ameloblasts, shortened Tomes’ processes, and papillary layer cells demonstrating reduced endocytic and lysosomal activities, thus perpetuating the retention of enamel proteins in cells and the matrix. Similar feedback responses occur in *Ambn* ^*Δ5,6/Δ5,6*^ mice in the transition from secretory to maturation stages. Ameloblasts lose polarity and stratification of the enamel organ epithelium due to the lack of functional Ambn protein (Fukumoto et al., 2004). Only with the reconstitution of full-length Ambn, was functionality of ameloblasts restored and enamel formed physiologically (Chun et al., 2010).

Maturation stage ameloblasts of *Ambn* overexpressers were reduced in height and detached when adjacent to cysts. The maturation stage was entered late due to prolonged secretory and transition stages and exited earlier than the wild-type indicted by changes in the cell morphology, resembling the reduced enamel epithelium. In the reduced enamel epithelium, ameloblasts lose polarization and merge with papillary layer cells into cuboidal cells. This protective state is normally reached when the mineralization of enamel has concluded, and the eruption of the tooth into the oral cavity is imminent. When ameloblasts entered this stage early, the duration of the maturation stage was shortened, thereby possibly hampering the accumulation of calcium and phosphate ions necessary for building the mineral content. The deviations in timing of amelogenesis were most prominent in the presence of the highest Ambn concentration (*Ambn Tg* ^*+++/+++*^), possibly due to cumulative effects leading to the inability of ameloblasts to compensate for the dysregulation. Cysts found in the enamel organ may contain fluid exerting pressure on enamel organ cells against the solid surface of the enamel. The enamel organ cells may be squeezed and forced to shorten their height and detach.

### Demarcation in relationship to the basement membrane and endocytosis

The demarcation of hypomineralized opacities perpendicular to the enamel thickness follows Hunter–Schreger bands (Jalevik and Noren, 2000) and marks abruptly reduced hardness and elastic modulus compared to normal enamel (Mahoney et al., 2004). The increased protein content of the MIH enamel compared to sound enamel (Schulze, 1970; Jälevik et al., 2001; Fagrell et al., 2010; Farah et al., 2010) is inverse to the mineral content (Farah et al., 2010) and enamel rod thickness (Jälevik et al., 2005). In order to accomplish proper mineralization of enamel, large quantities of degraded peptides and ions have to be transported across the ameloblast cell membrane and basement membrane through endocytosis (Pham et al., 2017). The transport of ions to increase mineralization is subsequent to protein degradation and endocytosis (Bronckers et al., 2015). In *Ambn* overexpressers, the continuity of exchange of molecules between the cellular plasma membrane and the enamel matrix across the basement membrane is impaired, as marked by fewer endosomes (Rab5), reduced lysosomal activity (cathepsin D), and a weak basement membrane, insufficient to attach ameloblasts. Endocytosis through plasma membrane-bound vesicles requires physical contact between the cargo content and the plasma membrane. Detached ameloblasts present a disruption in the endosomal pathway, preventing the generation of endosomes. Further evidence of reduced endocytosis activity was noted in the absence of Ambn in the Golgi apparatus, typical for wild-type ameloblasts (Nanci et al., 1987; Nanci et al., 1987). In the event of *Ambn* overexpression, endocytosis is impaired in the presence of prolonged secretory and transition stages, a diminished basement membrane, and detached ameloblasts, precluding mineralization.

The basement membrane plays a central role in amelogenesis as it is located at the interface of the enamel matrix or enamel surface and the ameloblasts. The basement membrane of maturation stage ameloblasts is present at the apical membrane of ruffle-ended and smooth-ended cells (Nanci et al., 1987). It is organized in cords consisting of proteins and pores to allow the passage of small molecules between the matrix and cells, including peptides and ions (Sawada and Inoue, 1996; Sawada and Inoue, 2003; Fouillen et al., 2017). The compromised basement membrane in *Ambn* overexpressers (*Ambn Tg* ^*+++*^ and *Tg* ^*+++/+++*^) was not attaching maturation stage ameloblasts properly to the enamel surface; consequently, the continuity between the matrix and ameloblasts was disrupted. The regions of the retained matrix corresponded to the hypomineralized enamel and alternated with areas of resorbed matrix and normal mineralization of the enamel of mandibular incisors. Similarly, detached ameloblasts were observed in *WDR72* null mice with hypomature enamel (Wang et al., 2015). The periodicity of attached and detached basement membranes of *Ambn* overexpressing incisors may relate to ruffle-ended and smooth-ended plasma membranes and differential Ambn expression. Ameloblasts in the maturation stage modulate plasma membranes facing the enamel surface as ruffle-ended or smooth-ended (Smith, 1998). In ruffle-ended ameloblasts, endocytosis and ion transport are active, while in smooth-ended ameloblasts, these activities cease to rest for the next cycle (Takano and Ozawa, 1980). Smooth-ended ameloblasts detach from the enamel surface and contribute to hypomineralized enamel in *Ncxkx4* null mice lacking functional SLC24A4 Ca^2+^-transporters (Bronckers et al., 2015; Bronckers et al., 2017). The continuity across enamel and ameloblasts provided by the basement membrane is critical for exchanging degraded peptides and ions between them.

## Conclusion

We propose the *Ambn* overexpression mouse as a model for opaque, demarcated hypomineralized enamel. The enamel lesions in molars and incisors of these mice present fundamental features of MIH: demarcated defects, opaque color, and reduced mineral content spanning the width of the enamel, starting at the innermost enamel and exhibiting enamel breakdown at the DEJ. This animal model will assist in elucidating the pathogenesis of the opaque, demarcated hypomineralized enamel by analyzing the ameloblast biology prior to tooth eruption. In the secretory stage, Ambn accumulates at the mineralization front and innermost enamel as incompletely cleaved and unresorbed fragments. Ameloblasts receive signals to prolong the secretory and transition stages and to advance to reduced enamel epithelium too early, resulting in abbreviated maturation stages. The basement membrane of the maturation stage is diminished and fails to attach ameloblasts for executing endocytosis. Ameloblasts have the ability to distinguish between the Ambn concentration and Ambn cleavage products, suggesting finely tuned feedback mechanisms. Future directions should include the analysis of ameloblastin peptides and Mmp20 stratified by the enamel sublayer in teeth with demarcated enamel lesions and MIH.

## Data Availability

The original contributions presented in the study are included in the Supplementary Materials, and further inquiries can be directed to the corresponding author.
