# Supplementary material for: Overexpression of ameloblastin in secretory ameloblasts results in demarcated, hypomineralized opacities in enamel
Source: Front Physiol. 2024 Jan 11;14:1233391. doi: 10.3389/fphys.2023.1233391 (PMC10808694; doi:10.3389/fphys.2023.1233391)
Supplement: Supplementary file 1 [file DataSheet1.docx]

**Supplemental Material**

**Overexpression of ameloblastin in secretory ameloblasts results in demarcated, hypomineralized opacities in enamel**

Yong-Hee Patricia Chun*, Chunyan Tan , Omar Villanueva , Madeline E Colley, Travis J Quintanilla, Mohamed S Basiouny, Caldonia A. Hartel, Cameron S. Critchfield, Stephan BH Bach, Roberto J Fajardo , Cong-Dat Pham

- Correspondence: Yong-Hee Chun, chuny@uthscsa.edu

**Supplementary Figure S1**


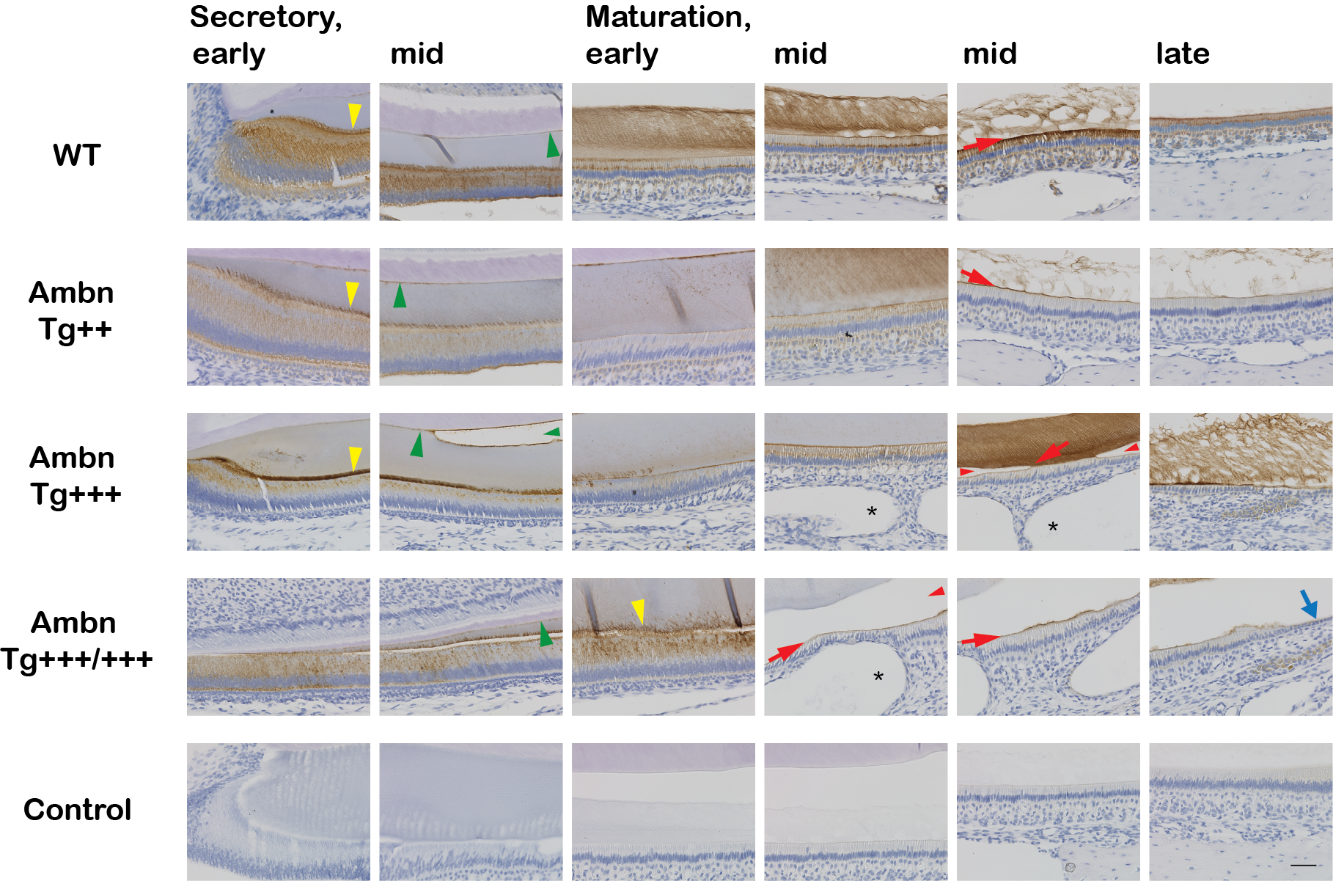


**Immunolocalization of Ambn in enamel organ epithelium.**

Mandibular incisors were sectioned sagittal and stages of enamel formation progress from secretory to maturation stages. In the wild-type, ameloblasts strongly expressed Ambn in the secretory stage with localization at the Tomes process and mineralization front (yellow arrowhead). At the maturation stage immuno-signals reduced in ameloblasts and localized to the Golgi apparatus in the subnuclear region, papillary layer and basement membrane. During early and mid-maturation stages, the enamel matrix displayed immuno-signals representing Ambn in the sheath. In *Ambn* overexpressing mice, Ambn immuno-signals accumulated in secretory vesicles (*Ambn Tg^+++/+++^*), at the mineralization front (*Ambn Tg^++^, ^+++^*) (yellow arrowhead), and at the dentino-enamel junction (DEJ) (*Ambn Tg^++^, ^++^*^+^) (green arrowhead). Compared to wild-type, immuno-signals were reduced in the maturation stage basement membrane (*Ambn Tg^++^, ^+++^, ^+++/+++^*) (red arrow). Cysts (asterisk) were present in the enamel organ epithelium in *Ambn Tg^+++^, ^+++/+++^*. Ameloblasts detached from enamel matrix (red arrowhead) in *Ambn Tg^+++^, ^+++/++^*^+^ and formed reduced enamel epithelium earlier compared to wild-type (blue arrow). Scale bar 50 μm.
